# Non-invasive electromagnetic fields induce anti-tumor neutrophil reprogramming and enhance checkpoint blockade in breast cancer

**DOI:** 10.3389/fimmu.2026.1861048

**Published:** 2026-10-16

**Authors:** Vivek Sharma, Chandra Khantwal, Kishori Konwar

**Affiliations:** 1 Asha Medical, San Ramon, CA, United States; 2 MuriGenics, Vallejo, CA, United States; 3 ScellAI, Revere, MA, United States

**Keywords:** electromagnetic field, endoplasmic reticulum stress (ER stress), immunotherapy, neutrophil reprogramming, triple negative breast cancer

## Abstract

**Introduction:**

Neutrophil reprogramming toward interferon-activated anti-tumor states represents an emerging mechanism of checkpoint sensitization; however, effective strategies to selectively induce this response in the tumor microenvironment remain elusive.

**Methods:**

We evaluated Asha Therapy, an investigational non-invasive, low-intensity (50 kHz, 2 mT) alternating magnetic field, in triple-negative breast cancer (TNBC) cells, integrating temporal transcriptomics, global proteomics, and cytokine profiling. CITE-seq profiling of tumor tissue was used to characterize the response of neutrophils and other cell populations in the tumor microenvironment. Therapeutic efficacy of Asha Therapy alone and in combination with anti-PD1 blockade was assessed in a murine TNBC model. A 19-gene neutrophil activation signature was derived from Asha-treated tumors and projected onto three independent human TNBC cohorts (METABRIC, n=348; SCAN-B, n=874; TCGA-BRCA, n=191), with multivariable Cox models adjusted for age, tumor stage, nodal status, and TNBC subtype.

**Results:**

Asha Therapy elicited a controlled endoplasmic reticulum (ER) stress response and adaptive unfolded-protein response in TNBC cells. This tumor-intrinsic stress matured from an acute signaling state into an immune-recruiting secretory program characterized by release of CXCL10, CCL2, and CCL20. These signals were associated with selective expansion and activation of neutrophils toward an interferon-responsive state, with additional transcriptional signatures observed within macrophage, T cell, and cancer-associated fibroblast populations. In the murine TNBC model, Asha monotherapy showed directional anti-metastatic effects, while combination with anti-PD1 blockade achieved significant therapeutic benefit, reducing lung metastasis and mortality by 88% (HR=0.12, p<0.05) without overt cytotoxicity. Projection of the 19-gene neutrophil activation signature onto the three human TNBC cohorts revealed significant association with improved overall survival (pooled HR=0.71, 95% CI: 0.60–0.83, p=0.01).

**Discussion:**

These findings indicate neutrophil reprogramming as a key feature of the low-intensity EMF therapeutic response. These preclinical findings support further investigation of Asha Therapy as a candidate non-pharmacologic strategy to activate innate immunity and improve responsiveness to checkpoint blockade in poorly immunogenic tumors.

## 1 Introduction

Cancer immunotherapy has transformed oncology by enabling durable tumor control through immune modulation (1). Immune checkpoint inhibitors targeting PD-1, PD-L1, and CTLA-4 have produced lasting responses in several malignancies, yet only a subset of patients benefit (2–9). Across most solid tumors, objective response rates remain 10–40%, and treatment is often limited by immune-related toxicities, with grade ≥3 events occurring in up to half of patients (10–13). Overcoming primary or acquired resistance while minimizing systemic toxicity remains a central challenge.

Triple-negative breast cancer (TNBC) exemplifies these challenges. Although pembrolizumab combined with chemotherapy improves outcomes in PD-L1+ TNBC, the majority of patients show minimal benefit with only 2–3 month progression free survival (PFS) gains, and treatment-related grade ≥3 toxicity occurs in nearly 30% of patients (9, 14). Furthermore, patients with visceral and central nervous system (CNS) metastases that are common in advanced TNBC were underrepresented or excluded from pivotal clinical trials, and retrospective data suggest poor immunotherapy responses in these sites (15, 16).

A key determinant of immunotherapy success is the modulation of the tumor microenvironment (TME) (17). In TNBC, checkpoint resistance is multifactorial, involving not only limited T cell infiltration but also accumulation of immunosuppressive myeloid populations and regulatory T cells, as well as the formation of stromal barriers that exclude or functionally impair effector lymphocytes (18–20). Numerous strategies have sought to reprogram the immunosuppressive TME, including radiation, oncolytic viruses, and targeted cytokine delivery, but these interventions often introduce additional toxicity or logistical complexity (21–23).

Neutrophils were historically considered exclusively pro-tumorigenic (20); however, recent evidence demonstrates that interferon-activated neutrophil states are associated with tumor control and enhanced checkpoint efficacy (24, 25). Transcriptional mapping of the neutrophil compartment has further defined a discrete IFN-response hub characterized by metabolic activation and anti-tumor function (26). Selective induction of this anti-tumor neutrophil phenotype therefore represents an emerging therapeutic opportunity, particularly in immunosuppressive tumors like TNBC where myeloid dysfunction is a dominant resistance mechanism (20).

Non-invasive physical modalities such as electromagnetic fields (EMF) represent a promising but underexplored means of immune activation. Novocure Ltd.’s tumor treating fields (TTFields) platform disrupts mitosis through alternating electric fields, is clinically approved across five tumor types, and recent preclinical studies indicate it can also induce immunogenic cell death and enhance antigen presentation (27–33). Similarly, the OncoMAGNETx device applies oscillating EMF to disrupt mitochondrial metabolism and elevate reactive oxygen species selectively in cancer cells, with early evidence of downstream immune effects in gliomas (34, 35). Most recently, it was demonstrated that low-intensity induced EMF reduce TNBC tumor growth and metastasis through immune modulation (36). However, these approaches rely primarily on cytotoxic mechanisms, and the molecular events linking EMF exposure to immune activation remain undefined. Whether low-intensity fields can reprogram the TME through non-cytotoxic adaptive stress, without direct cell killing, has not been investigated.

The present study was motivated by the discovery that cancer cells exposed to Asha Therapy, a low-intensity (2 mT, 50 kHz) alternating magnetic-field treatment (AMF), secreted inflammatory cytokines despite minimal direct cytotoxicity. This disconnect between robust molecular reprogramming and preserved cell viability suggests an adaptive stress response rather than a cytotoxic response. Thus, we hypothesized that ER stress acts as a candidate molecular bridge between EMF exposure and the immune response. This idea is based on three key observations: EMF can trigger stress pathways in mammalian cells (37–39), protein-folding stress is known to drive inflammatory signaling (40), and these resulting ‘danger signals’ have the potential to convert a suppressive TME into one that actively fights the cancer (41).

While the precise biophysical mechanism of 50 kHz fields remains under investigation, we focused this study on defining the biological translation of the physical stimulus. Using integrated transcriptomic, proteomic, and single-cell analyses, we found that Asha Therapy induced a controlled ER stress in cancer cells that is accompanied by selective expansion and activation of interferon-stimulated neutrophils in the tumor microenvironment. Additional transcriptional signatures were observed within the macrophage, cancer-associated fibroblast (CAFs) and T cell populations. Critically, in a murine TNBC model, combination with anti-PD1 blockade significantly reduced metastatic burden and mortality. To test whether the Asha Therapy-induced neutrophil program is conserved in human disease, we derived a 19-gene activation signature from Asha-treated murine neutrophils and projected it onto human TNBC patients across three independent cohorts, revealing significant association with improved overall survival. Together, these findings provide a preclinical rationale for EMF-induced immunomodulation, offer a framework for optimizing EMF parameters, and provide a rationale for potential therapeutic combinations with immunotherapies.

## 2 Materials and methods

### 2.1 Cell culture

MDA-MB-231 cells were cultured in RPMI-1640 medium (ThermoFisher #11835030) supplemented with 10% fetal bovine serum (FBS, ThermoFisher #A5209501) and 1% antibiotic-antimycotic solution (ThermoFisher #15240062). MDA-MB-468 cells were cultured in Dulbecco’s Modified Eagle Medium (DMEM, ThermoFisher # 11960044) supplemented with 10% FBS and 1% Antibiotic-Antimycotic solution. MCF-10A cells were cultured in specialized media (Elab Biosciences # CM-0525) supplemented with 10% FBS and 1% Antibiotic-Antimycotic solution. All cells were maintained at 37 °C in a humidified atmosphere with 5% CO_2_. Cell media was replaced every 2–3 days, and cells were typically passaged once they reached 50-80% confluence, and used within 20 passages.

### 2.2 Asha Therapy exposure

For *in vitro* experiments, Asha Therapy (50 kHz, 2 mT, sinusoidal waveform, 25% duty cycle) was delivered using a 75-mm radius Helmholtz coil placed inside a dedicated cell-culture incubator. The coil was designed to accommodate standard culture plates. Finite-element electromagnetic modeling (COMSOL Multiphysics) confirmed uniform EMF distribution across the plate area. Temperature was monitored using a fiber-optic thermocouple (Osensa PRB-100-1M-STM-MRI) placed in direct contact with the plate, and the incubator set point was adjusted to maintain 37.0 ± 0.5 °C throughout the experiment. A calibrated search coil was used to monitor field amplitude at the plate location. Therapy was delivered continuously for the full duration of each experiment. As the EMF would contaminate nearby plates, control samples were maintained in a separate but otherwise identical incubator. Environmental conditions (temperature, humidity, CO_2_, and plate format) were matched across both incubators, and control plates were handled in parallel with therapy plates to ensure identical timing of media changes, passaging, and sampling.

For *in vivo* experiments, Asha Therapy was delivered using a custom 185-mm radius Helmholtz coil designed to house a standard mouse cage housing 5–6 mice. The coil generated a uniform electromagnetic field that provided full-body exposure to all animals in the cage. A calibrated search coil was used to measure the magnetic field amplitude at the cage center to verify correct field delivery (± 10% of the target field). Therapy was applied continuously for the duration of the experiment unless otherwise specified.

Prior to the study, we measured the temperature differential between test and control cage locations and confirmed that Asha Therapy produced a temperature increase of less than 0.5 °C relative to control. Given this, a true field-off sham coil was not deemed necessary for this study, and control animals were instead housed in identical cages to the test animals, with similar handling across groups. Search coil measurements confirmed no detectable stray field at the control cage location. Both control and test cages were kept in the same room to ensure identical environmental conditions, including temperature, lighting, and humidity.

### 2.3 Thapsigargin

Thapsigargin (MedChemExpress #HY-13433) stock solution (500 μM) was prepared in DMSO (ThermoFisher # D12345) and stored at -80 °C. Working concentrations were prepared weekly by diluting the stock solution in culture medium to achieve the desired final concentration (IC_50_ for 72-hour exposure on MDA-MB-468 cells was previously determined to be 5nM).

### 2.4 *in vitro* Asha Therapy treatment

For Bulk RNA analysis, flow cytometery and cytokine measurements, 50,000 cells per well were seeded in 0.5 mL of culture medium in 24-well plates and incubated overnight at 37 °C. The following morning, thapsigargin (5 nM) was added to designated treatment groups as a positive control for ER stress induction. Plates were then placed either in a standard incubator (control and thapsigargin groups) or in the incubator equipped with the Asha Therapy setup for the designated therapy duration.

### 2.5 Bulk RNA sequencing

Following experimental treatments, culture supernatants were removed and cells were lysed using the Zymo Research RNA extraction reagent according to the manufacturer’s protocol. Total RNA was extracted and shipped to Novogene (Sacramento, CA, USA) for quality control, library preparation, and sequencing. RNA integrity was assessed using an Agilent 2100 Bioanalyzer.

Messenger RNA was purified using poly-T oligo-attached magnetic beads and fragmented prior to library construction. Libraries were prepared using a standard unstranded mRNA-seq workflow with random hexamer priming for first-strand cDNA synthesis, followed by second-strand synthesis (without dUTP marking), end repair, A-tailing, adapter ligation, size selection, and PCR amplification. Sequencing was performed on an Illumina NovaSeq X Plus platform to generate 2×150 bp paired-end reads.

### 2.6 Bulk RNA sequencing analysis

Raw paired-end FASTQ files were quality-filtered and adapter-trimmed using fastp (v0.23.2) (42). Cleaned reads were aligned to the human reference genome GRCh38 using STAR (v2.7.10a) with --quantMode GeneCounts and Ensembl gene annotations (release 113). Strandedness was evaluated from the STAR ReadsPerGene.out.tab files by comparing forward/reverse read ratios (mean = 1.06), confirming an unstranded library type; therefore, read counts were extracted from column 2 (unstranded) of each output file (43). Differential expression analysis was conducted using PyDESeq2 (version 0.5.2) with default parameters (44, 45). Genes with adjusted p < 0.05 (Benjamini–Hochberg correction), |log_2_ fold change| > 0.58, and baseMean > 10 were considered significantly differentially expressed.

For gene set enrichment analysis (GSEA) (GSEApy version 1.0.4), we started from PyDESeq2 outputs and ranked genes by the Wald statistic (t-like score) (46). Prior to ranking, we applied the following filters/normalization: (i) excluded genes with baseMean < 10; (ii) excluded rows lacking a valid HGNC gene symbol; (iii) collapsed duplicate symbols (multiple Ensembl IDs mapping to the same symbol) by retaining the entry with the largest |Wald statistic|; and (iv) removed rows with missing Wald statistics. Symbols were standardized case-insensitively to ensure uniqueness. GSEA was run against MSigDB Hallmark 2020, KEGG 2021 (Human), Reactome 2022, and GO Biological Process, Cellular Components, and Molecular Function gene sets, using the 15–500 gene set-size filter (47). Pathways with FDR < 0.05 were considered significant; positive/negative enrichment corresponded to higher/lower expression in Asha vs. control.

### 2.7 Flow cytometry

Following treatment exposure, cells were detached using trypsin-EDTA (Sigma Aldrich #59428C-500ML) and processed for flow cytometry analysis. Fc receptor blocking was performed using Fc receptor binding inhibitor antibody (ThermoFisher #14-9161-73) according to manufacturer’s instructions. Cell viability was assessed using fixable viability dye (ThermoFisher #65-0866-14). Cells were then fixed and permeabilized using fixation/permeability kit per manufacturer’s protocol (BD Biosciences Cytofix/Cytoperm™ Kit #554715).

Intracellular staining was performed using the following primary antibodies: GRP78-Alexa Fluor 647 (ThermoFisher #CL647-66574), CHOP-PE (Santa Cruz Biotechnology #sc-56107 PE), ATF4 (Santa Cruz Biotechnology #sc-390063 AF594), and XBP1s-Alexa Fluor 488 (ProteinTech #CL488-24868). Flow cytometry acquisition was performed on an Invitrogen Attune NXT flow cytometer, and data were analyzed using *De Novo* software (FCS Express). Single-stain controls were prepared for each fluorophore and used to calculate compensation matrices in FCS Express. Unstained cells served as negative controls. Flow cytometry gating is detailed in **Supplementary Figure S1**.

### 2.8 Cell viability assay

MDA-MB-468 cells (5,000 cells in 200 μL media) were plated overnight in black 96-well cell culture plates. The following day, media was replaced with fresh media, and the plates were then placed either in a standard incubator (untreated control group) or in the incubator equipped with the Asha Therapy setup for the designated therapy duration. After therapy exposure, cell viability was measured using PrestoBlue™ Cell Viability Reagent (ThermoFisher #A13261) per the manufacturer ‘s instructions. Briefly, the cell media was removed from each well and replaced with 100 μL of cell media containing 10% PrestoBlue reagent. Two wells on each plate containing media with 10% PrestoBlue reagent but no cells served as blanks for background subtraction. The plates were then placed in a standard incubator for 10 minutes, and the fluorescence was measured on a Thermofisher Varioskan LUX plate reader using an excitation wavelength of 560 nm and an emission wavelength of 590 nm. Fluorescence values were background-subtracted using the blank wells.

### 2.9 Cytokine analysis

Culture supernatants were collected from MDA-MB-468 cells at 24, 48, 72, and 96 hours following Asha Therapy exposure, immediately frozen, and stored at −80 °C until analysis. Samples were shipped on dry ice to Nomic Bio (Montreal, QC, Canada) for quantitative profiling using the nELISA platform, which measured 270 cytokines simultaneously with high sensitivity and multiplex precision (48). Asha-treated samples were compared with matched control samples at each time point using the Mann–Whitney U test. Multiplicity was corrected using the two-stage step-up False Discovery Rate (FDR) procedure of Benjamini, Krieger, and Yekutieli (Q = 5%), and cytokines with a false discovery rate (FDR) < 0.05 were considered significantly altered.

### 2.10 Proteomic analysis

Following experimental treatments, cells were washed with PBS, pelleted, and shipped on dry ice to PTM Bio (Alameda, CA, USA) for quantitative proteomic analysis. Proteins were extracted in SDS lysis buffer (8 M urea, 5% SDS, protease inhibitors, 50 mM TEAB, pH 8.0), quantified by BCA assay, reduced, alkylated, and digested using the Suspension Trapping (S-Trap) protocol. Peptides were analyzed by LC-MS/MS in Data-Independent Acquisition (DIA) mode on a Thermo Orbitrap Astral mass spectrometer coupled to a Vanquish Neo UHPLC system with a 15 cm × 100 µm C18 column. Samples were separated using a 23-minute gradient (4-99% acetonitrile with 0.1% formic acid) at 0.4-0.9 µL/min. Full MS scans were acquired at 240,000 resolution (m/z 380-980, AGC 500%, 3 ms max IT), with DIA MS/MS using 2 m/z isolation windows (m/z 150-2000, 25% collision energy). Raw data were processed using DIA-NN (v1.9) against the UniProt human reference proteome. Peptide and protein identifications were filtered at 1% false discovery rate (FDR), with label-free quantification based on DIA peak intensities.

Differential protein abundance was analyzed using DESeq2 (via pyDESeq2), which has been validated for mass spectrometry-based proteomics and handles the discrete count-like nature of peptide identification while accounting for technical and biological variability (49). Analysis was restricted to proteins detected in ≥2 samples per group to ensure reliable quantification. Proteins with padj<0.05 (Benjamini-Hochberg FDR correction) and |log2FC|>0.32 were considered significantly altered. GSEA was performed on reliably quantified proteins (≥2 samples per condition, n=8,534) ranked by sign(log_2_FC) × -log_10_(P-value), using MSigDB Hallmark, Gene Ontology (GO) and KEGG gene sets (FDR<0.05).

### 2.11 4T1 lung metastasis study

Female BALB/c mice (5–6 weeks old) were inoculated orthotopically with 500,000 4T1 tumor cells in the mammary fat pad, to model aggressive metastatic disease. Mice were housed in the Murigenics’ (Vallejo, CA) animal facility under standard conditions (12-hour light/dark cycle, 22 ± 2 °C) with Laboratory Rodent Diet and municipal tap water provided *ad libitum*. Tumor establishment was confirmed at 7 days post-inoculation by palpable tumor formation (average volume ~100mm³), and mice were randomized into four groups (n=6 per group) based on tumor size to ensure comparable baseline volumes between groups (**Supplementary Figure S2A**): isotype control, Asha monotherapy, anti-PD-1 monotherapy, and combination therapy. Mice were weighed twice weekly, and tumor measurements were performed by a trained technician using a digital caliper. Tumor volume was calculated using the formula (length * width * height)/2.

Asha Therapy was delivered continuously for the study duration. Isotype control antibody (250 μg, InVivoMAb rat IgG2a isotype control) or anti-PD-1 antibody (250 μg, BioXcell RMP1-14) was administered intraperitoneally twice weekly for a total of 8 doses. At day 28 post-randomization, mice were euthanized, via CO_2_ asphyxiation (4–5 L/min in a 7-liter cage) followed by cervical dislocation in accordance with IACUC guidelines. Lungs were harvested, and metastatic nodules were quantified by a trained technician blinded to the group assignment.

Statistical analysis was conducted using Kruskal-Wallis test followed by Dunn’s multiple comparisons test. Statistical comparisons were limited to hypothesis-relevant contrasts (monotherapy vs control, combination vs control, and combination vs monotherapy) to maintain statistical power.

### 2.12 4T1 survival study

Female BALB/c mice (5–6 weeks old) were inoculated orthotopically with 500,000 4T1 tumor cells in the mammary fat pad. Tumor establishment was confirmed at 7 days post-inoculation by palpable tumor formation (average volume ~100mm³), and mice were randomized into three groups based on tumor size to ensure comparable baseline volumes between groups: control (n=12), anti-PD-1 monotherapy (n=12), and combination of Asha Therapy and anti-PD1 (n=18) (**Supplementary Figure S2B**): Asha monotherapy was not included as prior experiments had demonstrated superior results with combination therapy. Asha Therapy was delivered continuously for the study duration, and anti-PD-1 antibody (250 μg, BioXcell RMP1-14) was administered intraperitoneally twice weekly for a total of 8 doses. 8 days after starting therapy, the primary tumor was resected under anesthesia to model post-surgical metastatic disease, and therapies were paused for a day to allow recovery. Animals were anesthetized using ketamine-xylazine (100 mg/kg ketamine + 10 mg/kg xylazine) and maintained on 1-1.5% isoflurane in oxygen delivered via nose cone throughout the surgical procedure. The tumor site was prepared by scrubbing with ethanol followed by betadine. An incision was made around the tumor site, the tumor was resected, and the skin was closed with silk sutures. Post-operative analgesia was provided with buprenorphine (0.1 mg/kg) administered for 72 hours following the procedure. Post-surgery wellbeing was monitored, and their weight was measured twice every week. Mice were sacrificed if their weight decreased or lab staff noticed significant health deterioration. Animal survival was monitored for 107 days after surgery, and the remaining mice were euthanized via CO_2_ asphyxiation (4–5 L/min in a 7-liter cage) followed by cervical dislocation in accordance with IACUC guidelines.

### 2.13 Cellular indexing of transcriptomes and epitopes by sequencing on tumor tissue

Female BALB/c mice (6–8 weeks old) were inoculated orthotopically with 100,000 4T1 tumor cells in the mammary fat pad. All animal procedures were approved by Murigenics’ Institutional Animal Care and Use Committee and conducted in accordance with NIH guidelines. Mice were housed in the Murigenics’ (Vallejo, CA) animal facility under standard conditions (12-hour light/dark cycle, 22 ± 2 °C) with Laboratory Rodent Diet and municipal tap water provided *ad libitum*. Tumor establishment was confirmed at 10 days post-inoculation by palpable tumor formation (average volume ~100mm³), and mice were randomized into two groups (n=6 per group) based on tumor size to ensure comparable baseline volumes between groups: untreated control and Asha Therapy. Following seven days of treatment, tumor volumes were measured using digital calipers, and mice were euthanized via CO_2_ asphyxiation (4–5 L/min in a 7-liter cage) followed by cervical dislocation in accordance with IACUC guidelines. Tumor tissues were immediately excised, submerged in pre-chilled MACS Tissue Storage Solution (Miltenyi Biotec #130-100-008), and stored at 4 °C during transport to MedGenome (Foster City, CA, USA). Three representative tumors from each group (n=3 per condition) were processed for CITE-seq analysis, consistent with published single-cell tumor microenvironment studies (24). Given this sample size, CITE-seq findings in this study are treated as exploratory and hypothesis-generating.

Single-cell suspensions were prepared from fresh tumor tissues using enzymatic and mechanical dissociation (MACS mice tumor dissociation kit, Miltenyi Biotec, per manufacturer’s instructions). Cell viability and concentration were assessed, and viable single cells were stained with the TotalSeq™-B Mouse Universal Cocktail (BioLegend) targeting 102 cell surface antigens, including principal immune lineage markers and 7 isotype controls. Cells were then processed for library preparation using the 10x Genomics Chromium Single Cell 3’ v3.1 Dual Index Gene Expression kit according to manufacturer’s protocol (50). Cells were partitioned into Gel Bead-In-Emulsions (GEMs) for barcoding, followed by reverse transcription, cDNA amplification, and library construction. Paired-end sequencing was performed on an Illumina NovaSeq 6000 platform with a target sequencing depth of approximately 50,000 reads per cell for gene expression (GEX) and 10,000 reads per cell for surface protein (ADT) detection.

Raw sequencing data were processed using Cell Ranger Count v7.1.0 (10x Genomics) for alignment to the mouse reference genome (mm10, 2020-A), barcode processing, and gene expression quantification. Quality control metrics confirmed high-quality data with Phred scores >30 across all read lengths. Preprocessing was performed using CellBender to remove ambient RNA contamination, followed by quality control filtering in Scanpy to retain cells with 200–4000 genes, <10% mitochondrial content, and <15% ribosomal content (51, 52). Doublets were removed using Scrublet, and genes expressed in <10 cells were excluded (53). Count data were normalized (library size = 10,000) and log-transformed. Integration of RNA and antibody-derived tag (ADT) data with batch correction was performed using TotalVI (54). UMAP dimensionality reduction and Leiden clustering at resolution 1.0 identified distinct cell populations (55, 56). Cell types were annotated based on RNA canonical marker gene expression and differential expression analysis and confirmed with ADT markers.

Compositional changes were quantified using scCODA, which models cell-type proportions in a Bayesian framework and accounts for the compositional nature of single-cell data, with automatic selection of reference cell type (57). Statistical credibility was assessed using default FDR threshold of 0.05. Differential expression analysis was performed using the following pseudobulking approach. For gene expression, raw UMI counts (for RNA) were summed per gene across all cells of a given cell type within each biological replicate, yielding a pseudobulk count matrix. DESeq2 (via pyDESeq2) was then applied to these pseudobulk profiles. This approach accounts for both within-sample (technical) and between-sample (biological) variability, avoids pseudoreplication, and provides superior control of false positives compared with single-cell-level Wilcoxon or t-tests (58–60). For ADT protein measurements, differential abundance was assessed using the Wilcoxon rank-sum test with Benjamini-Hochberg correction for multiple testing. GSEA was performed using GSEApy with MSigDB Hallmark 2020, KEGG 2019 Mouse, Reactome 2022, and Gene Ontology databases (FDR q<0.05).

To assess chromosomal instability in tumor epithelial cells, we calculated copy number variation (CNV) scores based on chromosome-level gene expression variance. Gene chromosomal coordinates were obtained from Ensembl BioMart (Mus musculus GRCm38/mm10, release 102). Genes were mapped to chromosomes 1-19, X, Y, and MT, with 20,740 genes (95.3% of detected genes) successfully annotated with genomic coordinates. For each cell, mean normalized gene expression was calculated per chromosome for autosomes 1–19 and chromosome X. Chromosomal heterogeneity scores were computed as the coefficient of variation (CV) across chromosomes:


CV score=σ(chromosome expression)/μ(chromosome expression).

Higher CV scores reflect greater inter-chromosomal expression variance, which correlates with chromosomal copy number alterations in aneuploid cells. While this approach does not resolve specific copy number changes, it provides a continuous metric to distinguish malignant from non-malignant epithelial populations based on chromosomal expression instability patterns (61).

### 2.14 Neutrophil activation signature derivation

To construct a human-projectable neutrophil activation signature, differentially expressed genes from Asha-treated tumor-infiltrating neutrophils were ranked by DESeq2 Wald statistic. From the top 100 upregulated transcripts, we excluded ribosomal, mitochondrial, and mouse-specific Gm loci and retained genes mapping to interferon/antiviral pathways, antigen-processing machinery, and treatment-induced myeloid activation. Mouse transcripts without direct human orthologs were replaced with their closest human equivalents (e.g., Gm16337 → IFNL3). This curation yielded a 19-gene “neutrophil activation signature” used for survival analyses. Full details of gene selection, and filtering criteria, are provided in **Supplementary Material S1**.

### 2.15 NeuMap hub module score comparison

NeuMap reference hub module scores were compared between treatment groups using a linear mixed-effects model with tumor sample included as a random intercept, applying small-sample degrees-of-freedom corrections (Satterthwaite approximation and Kenward-Roger correction) to account for the limited number of biological replicates (26). Analysis was performed in R using the lme4, lmerTest, and pbkrtest packages.

### 2.16 Prognostic association of the neutrophil activation signature in human TNBC

To test whether this signature is prognostic in human TNBC, we projected it onto bulk tumor transcriptomic data from three independent patient cohorts: METABRIC (n=348 patients that has been previously annotated as TNBC (62), data downloaded from cbioportal.org/study/summary?id=brca_metabric), SCAN-B (n=874 TNBC patients, data downloaded from data.mendeley.com/datasets/yzxtxn4nmd/4) and TCGA-BRCA (n=191 patients previously annotated as TNBC (62), data downloaded from https://www.cbioportal.org/study/summary?id=brca_tcga_pan_can_atlas_2018). Signature gene coverage varied by platform: 18 of 19 genes were available in METABRIC (*MNDAL* not detected) and SCAN-B (*FILIP1L* not detected), and 16 of 19 genes were available in TCGA-BRCA (*CMSS1*, *MNDAL*, and *IFNL3* not detected).

For each patient, we calculated signature scores as the mean normalized expression (z-scores) of the available signature genes. The SCAN-B dataset includes multiple sequencing libraries for a subset of patients and where duplicate libraries were present, signature scores were averaged at the patient level prior to analysis. Patients were stratified into high and low signature groups using median split, and overall survival was analyzed using Kaplan-Meier curves and the log-rank test. For each cohort, we fit Cox proportional hazards models to evaluate the association between high vs low neutrophil activation signature score and overall survival. Following a previously published approach (63), models were adjusted for nodal status (N1+ vs N0) and tumor stage (T2+ vs T1), with age (>50 vs ≤50 years) included as a stratification factor to account for potential non-proportional hazards. We additionally adjusted for TNBC intrinsic molecular subtype (basal vs non-basal), given its potential to confound the observed association. Tumor purity was not included as a covariate given inconsistent purity estimation methodology across cohorts (genomic purity available for METABRIC and TCGA-BRCA; SCAN-B, RNA-seq only, lacks an equivalent metric). The TNBC molecular subtype was extracted using the variable ‘CLAUDIN_SUBTYPE’ in METABRIC, ‘NCN.Subtype’ in SCAN-B, and ‘SUBTYPE’ in TCGA-BRCA.

Given that individual cohorts were estimated to have limited statistical power for multivariable-adjusted analysis, a meta-analysis across all three cohorts was conducted to assess the robustness and reproducibility of the observed associations. This meta-analysis was performed using random-effects model (Hartung-Knapp-Sidik-Jonkman method) to calculate pooled hazard ratios. Hazard ratios (HRs), 95% confidence intervals (CIs), and p-values were reported for the NSG score. All survival analyses were performed using the lifelines Python package (version 0.30.0). This computational approach to project cell-type-specific signatures onto bulk tumor transcriptomes follows established methodologies for myeloid and neutrophil lineage analysis in breast cancer (25, 63, 64).

## 3 Results

### 3.1 Bulk RNA sequencing reveals Asha Therapy induces ER stress and inflammatory response in TNBC cells

To characterize temporal dynamics of transcriptional response, we performed bulk RNA sequencing of MDA-MB-231 cells at 24 and 48 hours post-treatment. Principal component analysis (PCA) revealed minimal transcriptional divergence between Asha-treated and control cells at 24 hours, with clear separation emerging by 48 hours (**Figure 1A**).

**Figure 1:**
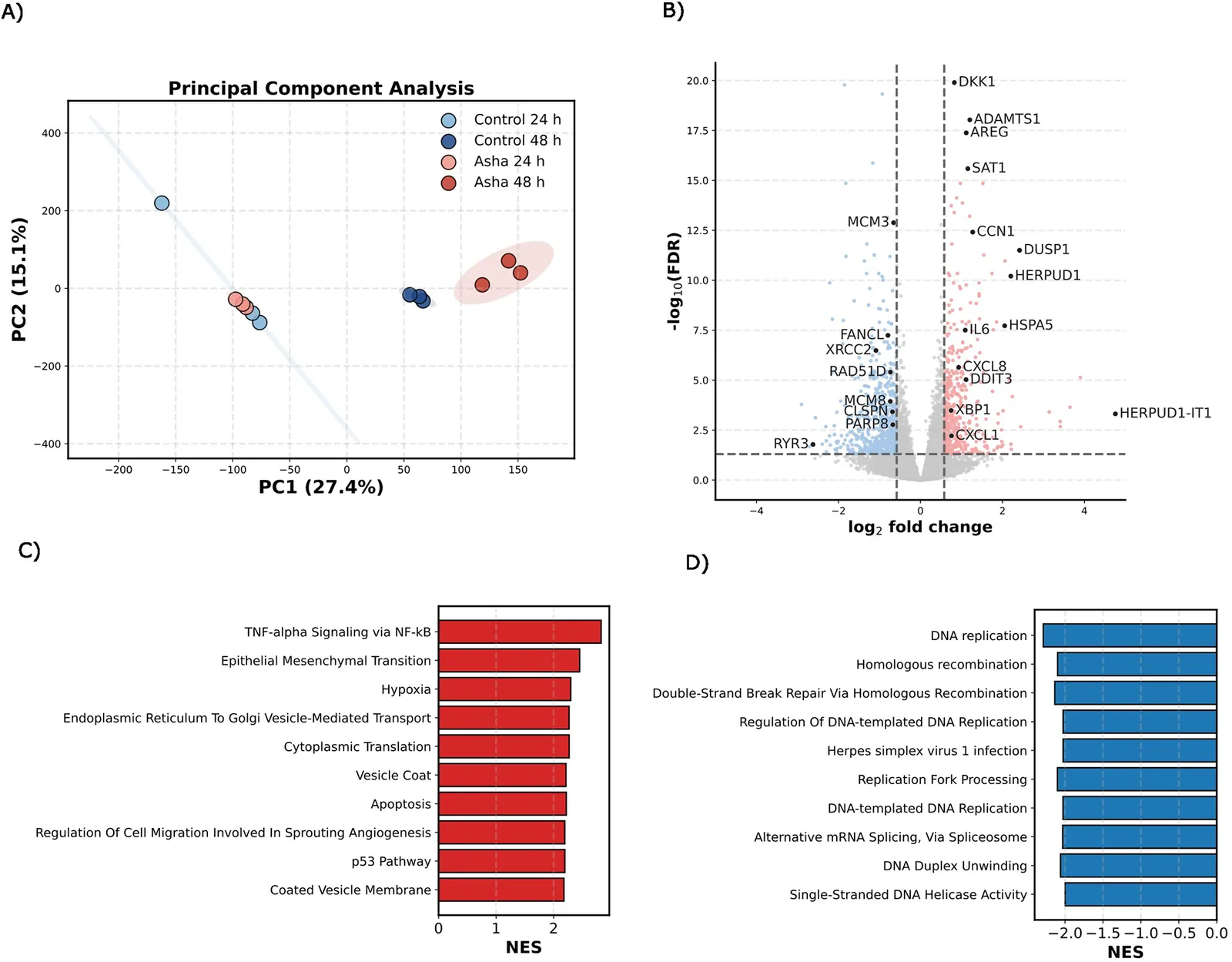
Bulk RNA sequencing reveals progressive transcriptional remodeling and multi-pathway activation following Asha Therapy. **(A)** Principal component analysis (PCA) of normalized gene expression profiles from MDA-MB-231 cells following Asha Therapy or control. Each point represents an independent biological replicate (n=3 per condition). Separation along PC1 and PC2 is evident by 48 hours, reflecting robust transcriptional changes at this time point. **(B)** Volcano plot displaying the differentially expressed genes (DEGs) in MDA-MB-231 cells following 48hours of Asha Therapy compared to control. In this plot, genes were filtered for an adjusted P value (Benjamini–Hochberg corrected) < 0.05, a |log2 fold change| > 0.58. Upregulated genes (red dots) are enriched for stress- and inflammation-associated signaling; downregulated genes (blue dots) are enriched for DNA replication and cell-cycle. a subset discussed in the Results is labeled, with the full list provided in **Supplementary Table S1**. **(C)** Gene Set Enrichment Analysis (GSEA) of up-regulated pathways in MDA-MB-231 cells at 48 hours, ranked by Normalized Enrichment Score (NES). GSEA identified 454 significantly up-regulated gene sets (FDR < 0.05), with the most significant enrichment corresponding TNF-alpha signaling via NF-kB, indicating activation of inflammatory transcriptional programs. Other major themes include ER-stress/UPR terms, ribosome biogenesis, and mitochondrial metabolism. **(D)** GSEA of down-regulated pathways in MDA-MB-231 cells at 48 hours, ranked by NES. GSEA identified 21 significantly down-regulated gene sets (FDR < 0.05). Down-regulated pathways are primarily related to cell-cycle regulation, DNA replication, and DNA repair, consistent with transcriptional repression of replication-associated genes at this time point. The full list of significantly upregulated and downregulated pathways is provided in **Supplementary Table S2**.

### 3.2 Differential gene expression

At 48 hours, the significantly (padj<0.05, |log_2_FC|>0.58, baseMean>10; **Figure 1B**; **Supplementary Table S1**) upregulated genes included the canonical ER-stress markers, including *HSPA5* (*GRP78*), *DDIT3* (CHOP), and XBP1. Inflammatory mediators including *IL6*, *CXCL8*/*IL8*, and *CXCL1* were also upregulated, as were stress-linked secreted factors including *AREG*, *DKK1*, and *ADAMTS1*. Multiple genes belonging to the DNA replication/repair and cell-cycle control were decreased, including *XRCC2*, *RAD51D*, and *FANCL*.

### 3.3 Gene set enrichment analysis

At 48 hours, the most significant (FDR < 0.05) enrichment corresponded to TNF-α signaling via NF-κB (NES = 2.74), indicating activation of inflammatory transcriptional programs. Other up-regulated pathways included multiple ER-stress and unfolded-protein-response terms (Hallmark UPR; GO and KEGG ER-stress modules) mirroring our DGE analysis, as well as ribosome biogenesis and protein synthesis, mitochondrial metabolism, and extracellular-matrix and cytoskeletal remodeling (**Figure 1C**; **Supplementary Table S2**). Significantly down-regulated pathways were primarily related to cell-cycle regulation, DNA replication, and DNA repair, consistent with transcriptional suppression of replication-associated genes at 48 hours (**Figure 1D**; **Supplementary Table S2**).

These data demonstrate that Asha Therapy is associated with coordinated ER stress and inflammatory signaling in TNBC cells while suppressing proliferative programs. The concurrent upregulation of ER stress markers and inflammatory cytokines is consistent with ER stress-associated immune signaling.

### 3.4 Unbiased global proteomic analysis reveals stress-induced metabolic remodeling in TNBC cells

To complement transcriptomic analysis and capture post-translational regulation, we performed unbiased global proteomic profiling of Asha-treated TNBC cells. Mass spectrometry-based label-free proteomic profiling of Asha-treated versus control MDA-MB-231 cells at 96 hours (selected to capture stable protein-level changes following the 48 hour transcriptional response) identified 8,882 proteins. PCA demonstrated clear separation between control and Asha-treated samples (**Figure 2A**), indicating distinct proteomic profiles.

**Figure 2:**
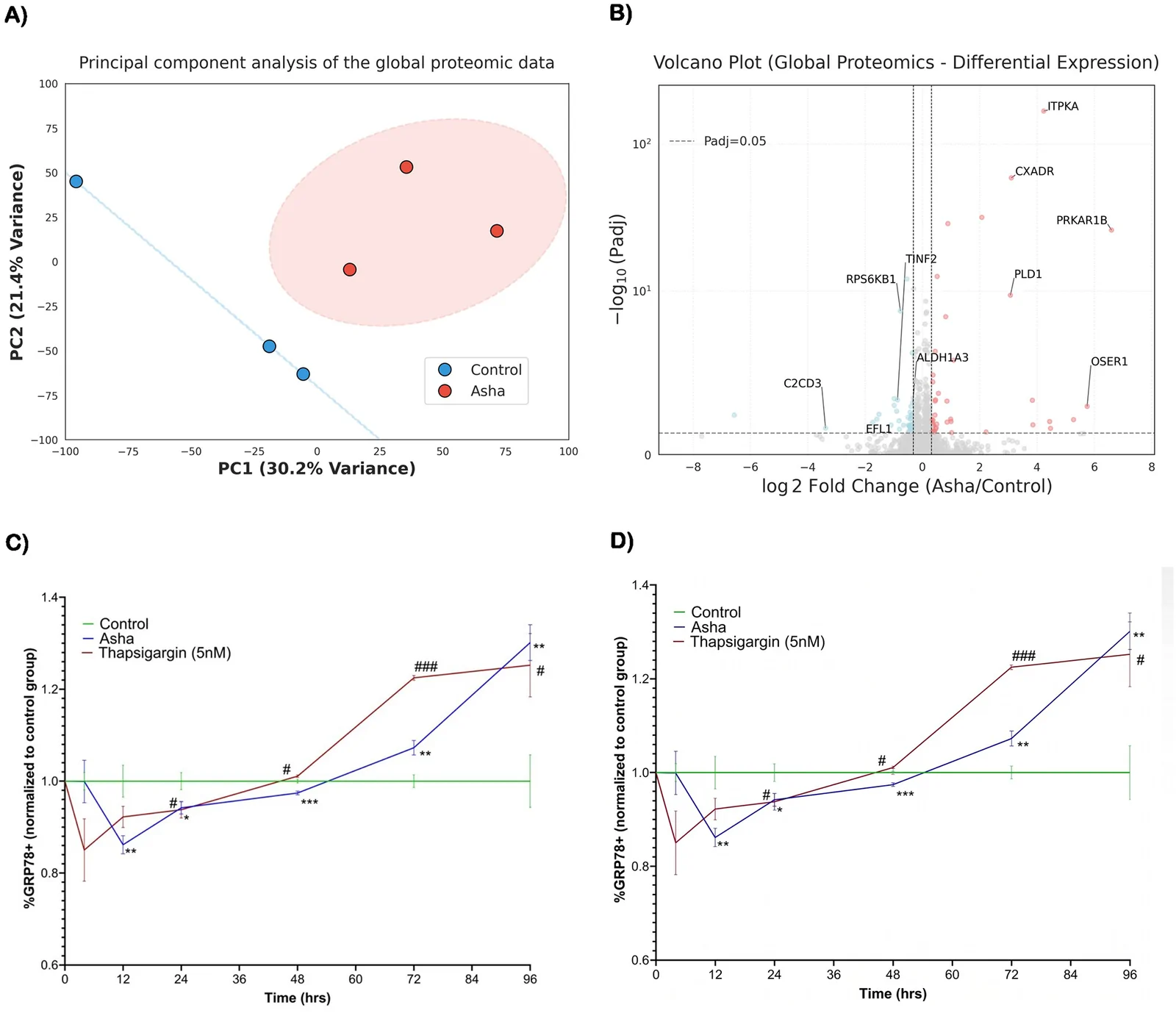
Asha therapy induces ER stress and proteome remodeling in TNBC cells. **(A)** Principal component analysis (PCA) of global proteomic profiles from control and Asha-treated MDA-MB-231cells at 96 hours. Each point represents an independent biological replicate (n=3 per condition). Clear separation indicates distinct proteomic responses to treatment. **(B)** Volcano plot of differentially expressed proteins in MDA-MB-231 cells at 96 hours post-Asha treatment. Differential protein abundance analysis between Asha-treated and control samples (n=3 per group) was assessed using DESeq2. P-values were adjusted for multiple comparisons using the Benjamini-Hochberg procedure (FDR). Proteins were filtered for significance based on padj < 0.05 and |log2 fold change| > 0.32. A total of 88 proteins were significantly altered (43 upregulated, red dots; 45 downregulated, blue dots), a subset discussed in the Results is labeled, with the full list provided in **Supplementary Table S4**. Upregulated proteins are enriched for signaling, proteostasis, and inflammatory components, while downregulated proteins are associated with protein quality control and metabolism. **(C, D)** Time-course analysis of ER stress markers in MDA-MB-468 cells exposed to Asha therapy, thapsigargin (5 nM, positive control), or left untreated (control) for up to 96 hours. Levels of GRP78 **(C)** and XBP1s **(D)** were measured by flow cytometry. CHOP and ATF4 showed similar trends with more modest induction (**Supplementary Figures S3A, B**). Data are presented as mean ± SEM (n=6 per group per time point), normalized to the control group at each time point. Statistical significance was determined using multiple unpaired t-tests comparing each treatment group to the control group at each time point. Significance markers: *,^#^(p < 0.05); **,^##^(p < 0.01); ***,^###^(p < 0.001).

Differential analysis of proteins reliably detected in both conditions (≥2 samples per group), using a platform-appropriate threshold reflecting the established compression of protein-level fold-changes relative to transcript-level changes (padj<0.05, |log2FC|>0.32) (65), identified 88 significantly altered proteins (**Figure 2B**; **Supplementary Table S3**). Key upregulated proteins were primarily associated with ER stress and calcium signaling pathways, including ITPKA (18.9-fold), CXADR (8.6-fold), and PLD1 (8.4-fold). We also observed a marked induction of PRKAR1B (97-fold), a regulatory subunit of protein kinase A (PKA) that mediates stress-adaptive signaling. Conversely, downregulated proteins included the mTOR effector RPS6KB1, the cancer stemness marker ALDH1A3, and DNA maintenance factors such as TINF2 and EFL1. Additionally, 17 proteins were newly detectable following Asha Therapy (≥2/3 Asha samples, undetectable in controls), signifying the activation of previously undetected proteins (**Supplementary Table S4**). These included the ER-stress regulator NUPR1 and the nutrient-stress sensor ASNSD1.

Together, these findings indicate that Asha Therapy is associated with reduced expression of proliferative and stem-like markers characteristic of steady-state TNBC tumor cells, alongside a stress-adapted secretory profile. Induction of NUPR1 and PRKAR1B is consistent with activation of stress-adaptive secretory pathways. This proteomic profile is consistent with the ER stress and immune-recruiting secretory program observed at the transcriptomic level and the downstream immune infiltration observed in our *in vivo* studies.

### 3.5 Flow cytometry results indicate Asha Therapy induces ER stress and UPR activation in TNBC cancer cells

To characterize the temporal dynamics of ER stress responses, we performed a time-course analysis using the MDA-MB-468 TNBC cell line measuring ER stress marker GRP78 and UPR markers (XBP1s, CHOP and ATF4) (66, 67). We used MDA-MB-468 cells for the time-course experiment because their ER stress markers change over a broader range after stimulation, making it easier to track early and late phases of the UPR. Flow cytometry measurements were obtained 6, 12, 24, 48, 72, and 96 hours after Asha treatment (n=6/group/time point), and thapsigargin (5 nM, 72-hour IC50 value derived experimentally, a SERCA pump inhibitor that depletes ER calcium stores) was used as a positive control for ER stress (68).

Both Asha and thapsigargin treatment groups exhibited an initial decrease in GRP78, ATF4, XBP1s, and CHOP during the first 12–48 hours, followed by gradual recovery and elevation between 72–96 hours (**Figures 2C, D**; **Supplementary Figures S3A, B**). Notably, thapsigargin induced robust increases in all UPR markers by 96 hours, while Asha Therapy produced more modest elevations (significant in GRP78, XBP1s and CHOP), without reducing cell viability (**Supplementary Figure S3C**), and is broadly consistent with an adaptive rather than terminal ER stress response. This pattern aligns with our transcriptomic and proteomic data showing stress-adaptive signaling without activation of cell death pathways.

### 3.6 Cytokine analysis reveals Asha Therapy induces a temporal immune-recruiting secretory program in TNBC cells

ER stress is known to trigger secretion of pro-inflammatory cytokines and damage-associated molecular patterns that can activate immune responses (40). To characterize how Asha-induced ER stress translates into altered tumor secretory programs, we profiled 270 cytokines in conditioned media from MDA-MB-468 cells at 24, 48, 72, and 96 hours using the nELISA platform (48). Conditioned media showed temporal shifts in secreted factors (**Figures 3A–D**). Early responses (24h) included matrix remodeling enzymes (uPA, ADAMTS1), followed by transient inflammatory signals at 48h (IL-6, Tissue Factor). By 72-96h, a sustained immune-recruiting program emerged, with increased secretion of chemokines with known roles in myeloid recruitment and T cell chemoattraction, including CCL2, CXCL10, CCL20, and CXCL1 (69–71).

**Figure 3:**
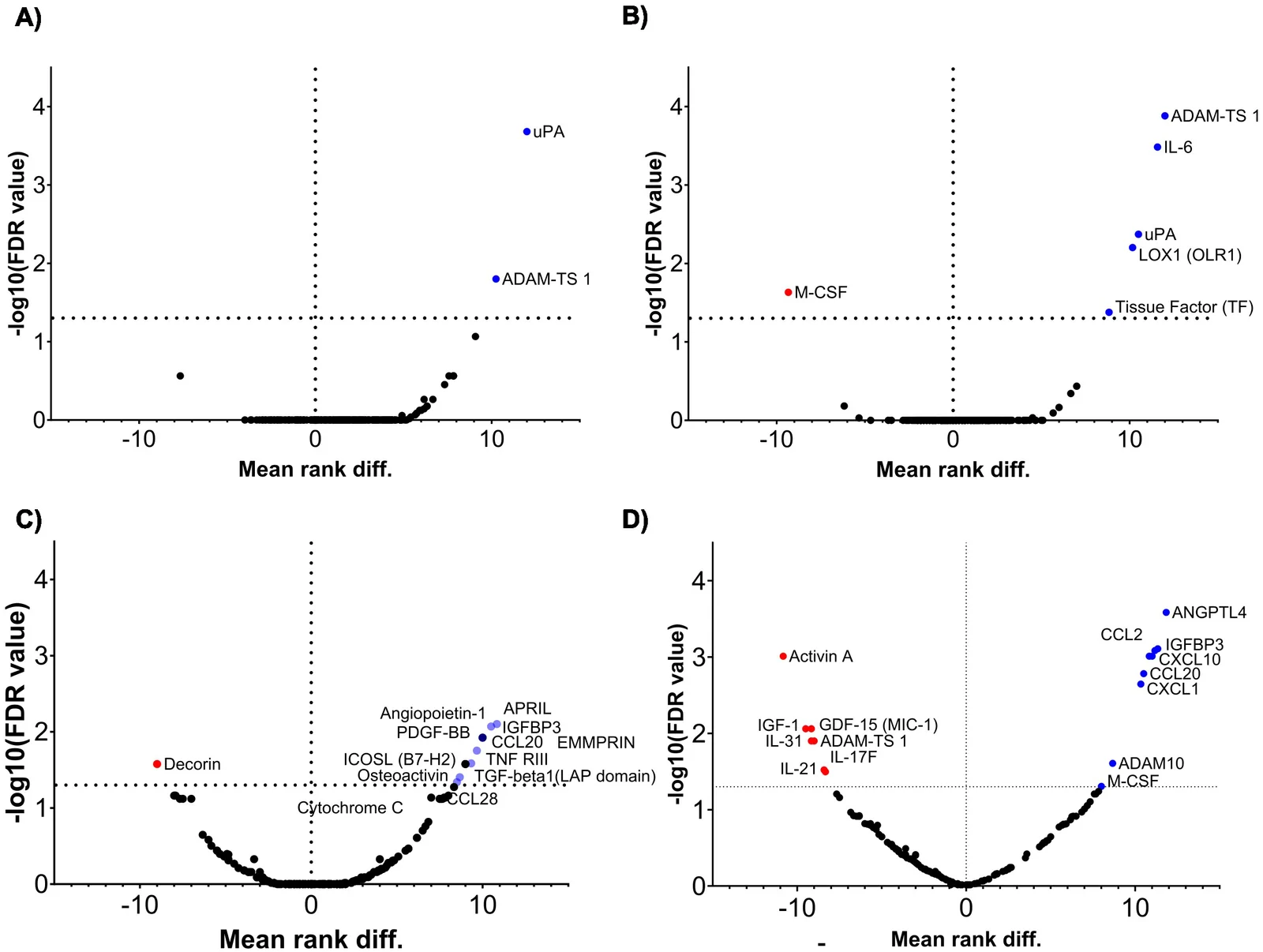
Temporal cytokine profiling reveals dynamic secretome remodeling in MDA-MB-468 cells following Asha therapy: Volcano plots display differential cytokine expression in culture supernatants from Asha-treated versus control cells at **(A)** 24 hours, **(B)** 48 hours, **(C)** 72 hours, and **(D)** 96 hours, analyzed using nELISA (270-cytokine panel). Statistical significance was determined using multiple Mann-Whitney U tests. Multiplicity was corrected using the two-stage step-up False Discovery Rate (FDR) procedure of Benjamini, Krieger, and Yekutieli (Q = 5%), and cytokines with a false discovery rate (FDR) < 0.05 were considered significantly altered. The x-axis represents mean rank difference (Asha – control), and the y-axis indicates −log_10_(FDR) values from Mann–Whitney testing. Cytokines above the horizontal dotted line met the significance threshold (FDR < 0.05). Early time points (24–48 hours) show selective upregulation of ADAMTS1, uPA, and IL-6, whereas later stages (72–96 hours) exhibit broader activation of inflammatory and chemotactic cytokines, including CCL2, CXCL10, CCL20, and CXCL1. The dashed vertical line denotes zero mean rank difference (no change). Each dot represents one cytokine analyzed (n=12 sample/condition/time point).

Notably, IL-6 and ADAMTS1 showed concordant upregulation at both transcript (bulk RNA-seq, 48h) and protein (secreted cytokine) levels. Chemokine secretion at 72-96h suggests that Asha-treated tumor cells develop a secretory program capable of attracting myeloid cells and T cells.

### 3.7 Asha Therapy reduced metastases and improved survival in conjunction with anti-PD1 in 4T1

The coordinated induction of ER stress markers and the subsequent emergence of a pro-inflammatory secretory program *in vitro*, characterized by the release of myeloid and T cell chemoattractants (CCL2, CXCL10, and CXCL1), suggested that Asha Therapy could remodel the immunosuppressive TNBC TME to improve responsiveness to immune checkpoint blockade. To test this hypothesis, we evaluated Asha Therapy in combination with anti-PD1 in the 4T1 orthotopic model.

An initial four-week study evaluated Asha Therapy’s anti-metastatic efficacy in a 4T1 (TNBC) orthotopic syngeneic immunocompetent model. Mice with established tumors were randomized into four groups (n=6/group): control, Asha monotherapy, anti-PD1 monotherapy, and combination therapy. At study completion, lung metastatic nodules were microscopically counted by a trained technician. While Asha monotherapy showed a directional reduction in metastatic burden that did not reach statistical significance, the Asha/anti-PD1 combination achieved significant reduction in lung metastases compared to control (adjusted p=0.037; **Figure 4A**). These effects occurred without significant changes in primary tumor volume (**Supplementary Figure S4A**), and the treatment was well-tolerated, with no weight loss observed in any experimental group throughout the study (**Supplementary Figure S4B**).

**Figure 4:**
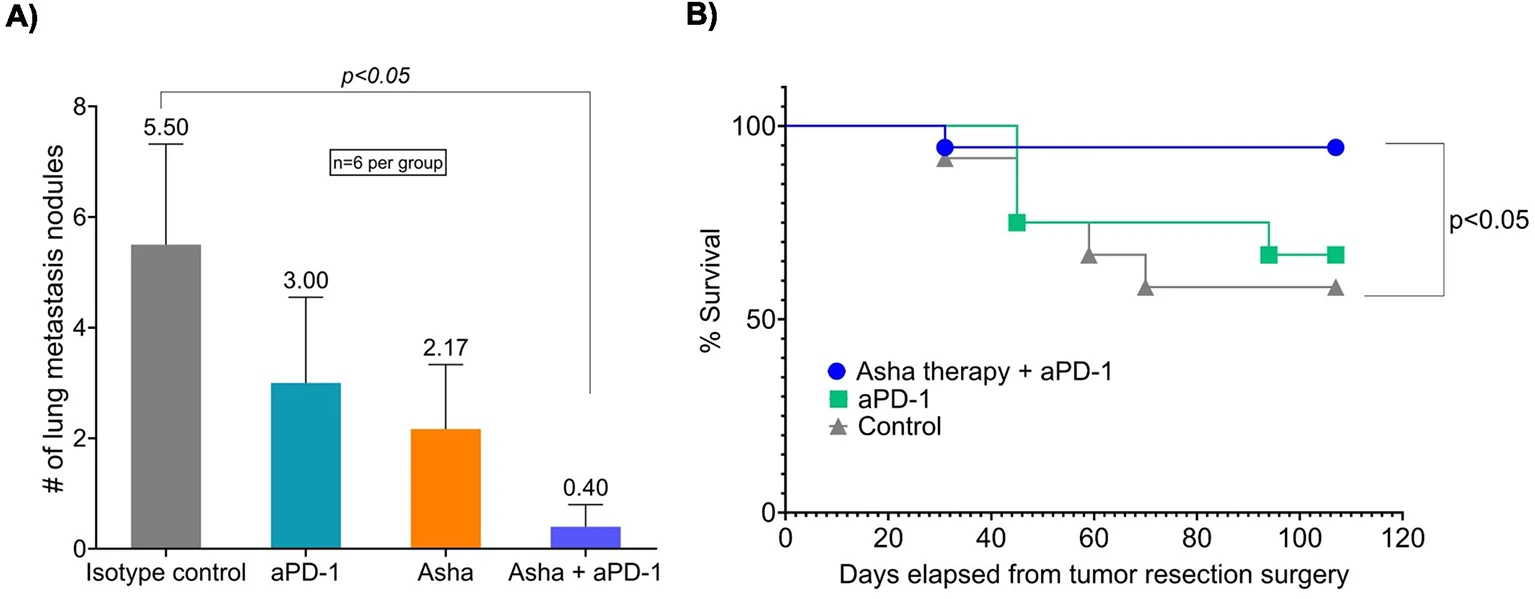
Asha therapy in combination with anti-PD1 significantly reduced metastatic burden, and improved survival in 4T1 TNBC model. **(A)** Lung metastasis quantification in 4T1 tumor-bearing mice following 4-week treatment. Female BALB/c mice with established orthotopic 4T1 tumors were randomized to control (n=6), Asha monotherapy (n=6), anti-PD1 monotherapy (n=6), or combination therapy (n=6). Metastatic nodules were microscopically counted at study completion. Data are shown as individual values with mean ± SEM. Statistical analysis by Kruskal-Wallis test followed by Dunn’s multiple comparisons test **(B)** Kaplan-Meier survival analysis following primary tumor resection. Female BALB/c mice with established 4T1 tumors received 1 week of treatment (control, n=12; anti-PD1 monotherapy, n=12; Asha + anti-PD1 combination, n=18), followed by surgical resection of the primary tumor. Mice were monitored for survival over 107 days post-surgery. Combination therapy significantly reduced mortality risk compared to control (HR = 0.12, 95% CI: 0.02–0.63, p<0.05, log-rank test).

A subsequent study using the 4T1 metastasis model further evaluated this combination effect. Immunocompetent mice with established tumors were randomized into three groups: control (n=12), anti-PD1 monotherapy (n=12), and Asha Therapy/anti-PD1 combination (n=18). After one week of treatment, the primary tumor was resected, and the mice were monitored for survival. Anti-PD1 monotherapy showed no significant survival benefit, while the combination therapy reduced death risk by 88% vs control (p<0.05, Hazard ratio = 0.12, 95% CI: 0.02-0.63; **Figure 4B**). Consistent with our earlier findings, longitudinal monitoring confirmed the safety of the combination regimen, with no evidence of treatment-induced weight loss (**Supplementary Figure S4C**). These findings demonstrate that Asha Therapy improves the response to anti-PD1 therapy in a model with limited monotherapy efficacy, significantly improving survival when combined with anti-PD1.

### 3.8 CITE-seq profiling reveals immune reprogramming in the tumor microenvironment

To characterize how the *in vitro* stress and secretory responses translate to the tumor microenvironment *in vivo*, we performed CITE-seq profiling of dissociated tumor tissues from control (n=3) and Asha-treated (n=3) mice at 7-days following treatment initiation. Single-cell analysis yielded a median of 4,487 high-quality cells per sample (range: 3,879–5,159). Unsupervised clustering identified 11 distinct populations, which were annotated based on canonical marker gene expression, and confirmed using surface protein markers (**Figures 5A–C**; **Supplementary Figure S5**). Subsequent analyses focused on the five most abundant cell types (epithelial/malignant cells, monocytes/macrophages, neutrophils, T cells, and fibroblasts), which collectively represented >90% of profiled cells. Six rare populations (dendritic cells, plasmacytoid dendritic cells, NK cells, endothelial cells, mast cells, and B cells) were catalogued but excluded from differential expression analysis due to insufficient cell numbers. These populations are characteristically rare in the 4T1 TNBC model (72), consistent with the poorly immunogenic nature of this tumor (73).

**Figure 5:**
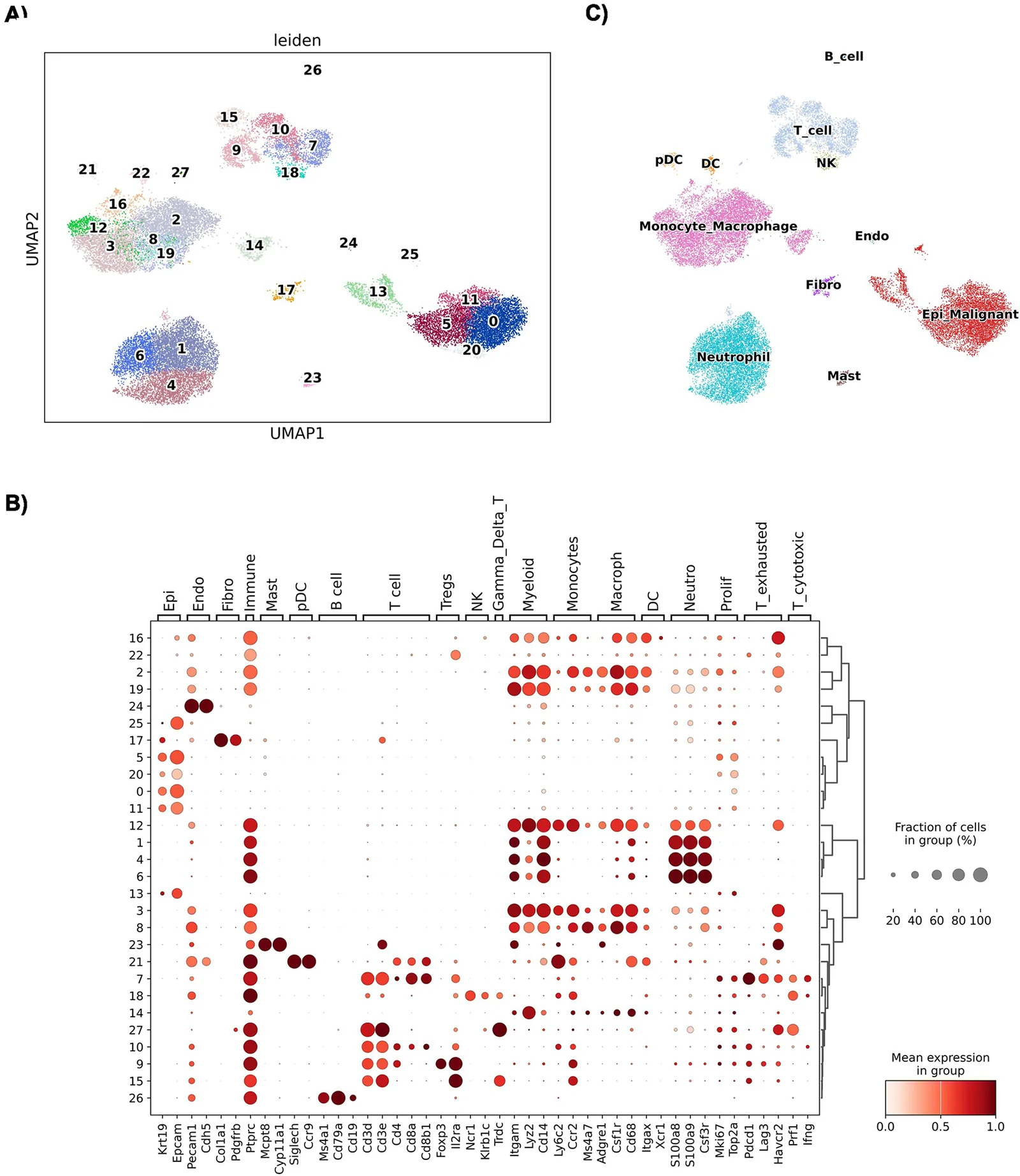
Cellular composition of the 4T1 tumor microenvironment. **(A)** UMAP of 26,446 tumor-infiltrating cells colored by Leiden cluster identity (28 clusters). **(B)** Dot plot showing expression of canonical lineage markers used for cluster identification; dot size indicates the fraction of cells expressing the marker, and color intensity represents mean expression level. **(C)** UMAP colored by annotated cell type (11 populations). Related Leiden clusters were merged based on shared lineage-specific marker expression.

### 3.9 Asha Therapy elicits translational and interferon-driven programs in malignant cells

To determine whether the stress responses observed *in vitro* persist in the complex tumor microenvironment, we analyzed malignant cell transcriptional states within Asha-treated tumors. Epithelial sub-clustering identified approximately 20% of cells as malignant based on high copy number variation, elevated G2M scores, and Mki67 expression (**Figure 6A**). Restricting differential expression to these high-confidence malignant cells (n=1,394) minimized stromal contamination.

**Figure 6:**
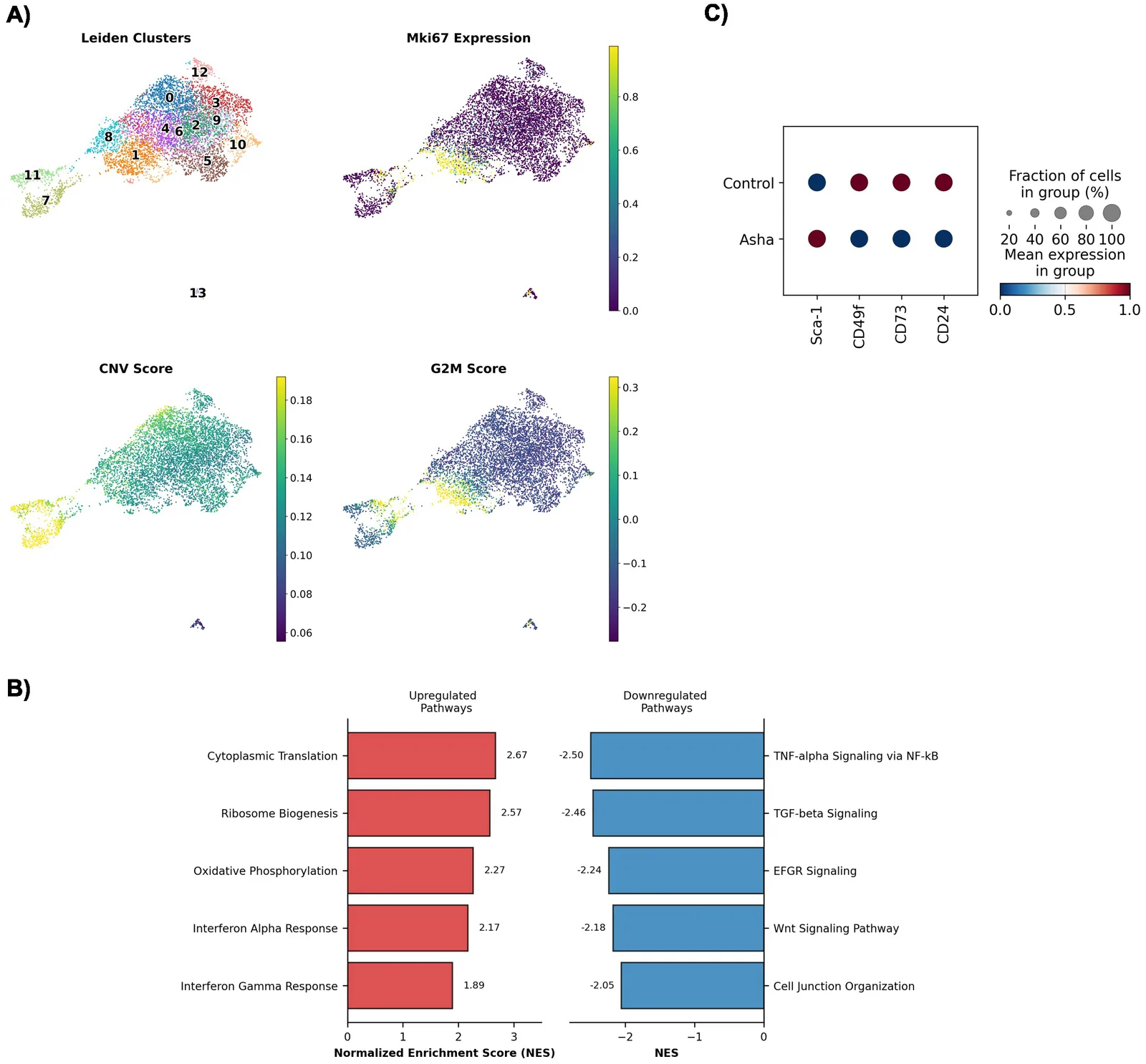
**(A)** UMAP plot of sub-clustered epitheilial cells displaying leiden sub-clusters, the proliferation marker Mki67, copy number variation (CNV) scores, andG2M cell cycle scores. Based on elevated Mki67, elevated CNV scores and G2M scores, leiden sub-clusters 0, 10, and 12 were identified as malignant cells. **(B)** Gene Set Enrichment Analysis (GSEA) of malignant cells comparing Asha-treated vs. Control tumors. Bar plot displays representative biological programs significantly enriched (FDR < 0.05) in treated cells, ranked by Normalized Enrichment Score (NES). Upregulated pathways (red) denote a shift toward enhanced biosynthetic and interferon-mediated activity. Downregulated pathways (blue) reflect reduced growth-factor and structural-organization pathway activity. The full list of significantly upregulated and downregulated pathways is provided in **Supplementary Table S6**. **(C)** Dot plot displaying CITE-seq protein expression for key interferon-response (Sca-1) and immune-evasive/stemness markers (CD49f, CD73, CD24) in the malignant cell compartment. Color indicates the relative mean expression (standardized per marker to highlight shifts), and dot size represents the percentage of cells expressing each marker. A full list of significantly expressed protein markers is provided in **Supplementary Table S7**.

Differential expression revealed upregulation of interferon-induced genes (*Ifi27l2a*, log_2_FC = 2.65) and stress-associated genes (*Filip1l*, log_2_FC = 1.44), alongside the immune checkpoint regulator *Cd274/PD-L1* (log_2_FC = 1.09). Downregulated genes included stress-responsive transcription factors (*Klf2*, log_2_FC = -1.17) (**Supplementary Table S5**). GSEA revealed upregulation of biosynthetic programs (cytoplasmic translation, NES = 2.67; ribosome biogenesis, NES = 2.57; oxidative phosphorylation, NES = 2.27; MYC targets, NES = 2.00) alongside interferon signaling (IFN-α, NES = 2.17; IFN-γ, NES = 1.89). Notably, downregulated pathways included TNF-α/NF-κB signaling (NES = -2.50), growth factor pathways (TGF-β, NES = -2.46; EGFR, NES = -2.24; Wnt, NES = -2.18), MAPK cascade (NES = -2.04), and cell junction organization (NES = -2.05) (all FDR < 0.05; **Figure 6B**; **Supplementary Table S6**).

Under continuous therapy, malignant cells exhibited interferon-driven and biosynthetic programs without persistent elevation of acute ER stress markers. This pattern is consistent with the initial ER stress response (documented at 48-96h *in vitro*) resolving prior to this later timepoint, with interferon and metabolic activity observed alongside it. At the protein level, CITE-seq showed interferon pathway activation with increased surface expression of the interferon-stimulated marker Sca-1 (log_2_FC = +0.43), alongside reduced expression of the immunosuppression marker CD73 (-0.54) and stemness markers CD49f (-0.93) and CD24 (-0.42), indicating enhanced immunological visibility and reduced immune evasion capacity (**Figure 6C**; **Supplementary Table S7**).

### 3.10 Asha Therapy is associated with transcriptional and surface marker changes in tumor fibroblasts

Given that our cytokine profiling revealed secretion of factors capable of remodeling the tumor stroma, we conducted an exploratory analysis of fibroblast transcriptional and surface marker changes following Asha Therapy. Differential expression showed downregulation of cytoskeletal genes (*Myh11*, *Mylk*, *Des*), matrix components (*Col4a1*, *Col18a1*), and *Il6*, alongside upregulation of stress-responsive genes (*Ankrd1*), the neutrophil-recruiting chemokine *Cxcl3*, and interferon-stimulated genes (*Ifit1*, *Ifi202b*) (**Supplementary Table S8**). GSEA (**Supplementary Figure S6A**; **Supplementary Table S9**) showed negative enrichment of epithelial-mesenchymal transition (NES=-2.19) and ECM organization (NES=-2.13), with positive enrichment of ribosomal (NES = 2.60), hypoxia (NES = 2.21), and interferon response pathways (NES = 2.05-2.00).

At the protein level, CITE-seq showed reduced expression of contractile surface protein markers, including Thy1/CD90.2 (log_2_FC = -0.63), and integrin subunits (CD49a, CD49d, CD61) that mediate matrix adhesion, alongside reduced expression of immune regulatory molecules (MHC-II, CD86, CD40, PD-L1) (**Supplementary Figure 6B**; **Supplementary Table S10**). Together, these exploratory observations suggest that Asha Therapy is accompanied by transcriptional and surface marker changes within cancer-associated fibroblasts.

### 3.11 Asha Therapy increases neutrophil infiltration and is associated with reprogramming of neutrophils toward an interferon-activated state

Neutrophils represent a critical myeloid population capable of both tumor-promoting and anti-tumor functions depending on their activation state. We therefore examined neutrophil responses to Asha Therapy. Compositional modeling (scCODA) identified neutrophils as the only immune population significantly increased following Asha Therapy (log_2_ fold-change = 0.81, FDR < 0.05), while other compartments remained unchanged (**Figure 7A**). This selective expansion is consistent with recent reports that effective immunotherapies induce proliferation of interferon-stimulated gene (ISG)^+^ anti-tumor neutrophils (24, 26).

**Figure 7:**
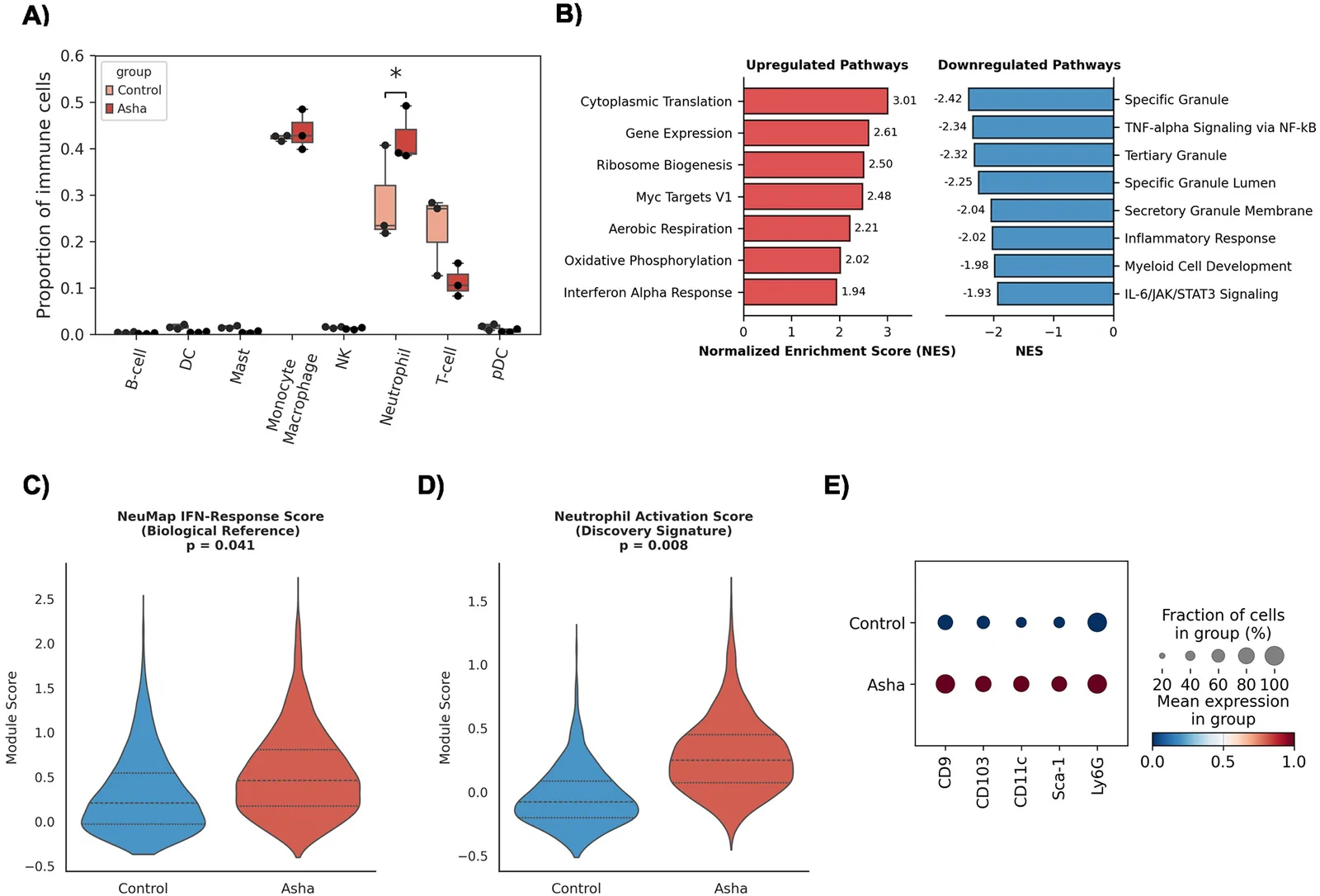
Asha therapy induces a specific interferon-responsive neutrophil activation state. **(A)** scCODA compositional analysis showing neutrophils as the sole significantly expanded immune population (log_2_FC = 0.81, FDR < 0.05). **(B)** GSEA of neutrophils showing upregulated metabolic/interferon pathways (left, red) and downregulated immunosuppressive/granule pathways (right, blue). NES values indicated on bars; all pathways shown have FDR < 0.05. **(C)** Violin plot of the NeuMap IFN-response module score, showing a shift toward the anti-tumor trajectory following Asha therapy. **(D)** Violin plot of the bespoke Neutrophil Activation Score derived from the top differentially expressed genes in this study, shown alongside panel C as a discovery-derived signature rather than an independent validation. **(E)** Dotplot showing relative expression of key activation and maturation markers following Asha therapy. Asha therapy is associated with an increase in the surface density of activation markers (CD9, CD103, CD11c) and the interferon-stimulated antigen Sca-1, with a concurrent reduction in the maturation marker Ly6G. Color indicates standardized mean expression per marker, and dot size represents the percentage of cells expressing each marker. For violin plots **(C, D)**, internal lines represent quartiles and the median, and statistical significance was determined using a linear mixed-effects model with tumor as a random intercept, applying Satterthwaite and Kenward-Roger small-sample corrections.

Differential gene expression analysis revealed that Asha Therapy exposure downregulated genes including azurophilic and specific granule components (*Ngp*, *Ltf*, *Camp*, *Mmp8*, *Mmp9*, *Lcn2*) and the immunosuppressive marker *Chil3*, while upregulating genes included T cell-recruiting chemokines (*Cxcl9*), and interferon-stimulated genes (*Ifit3*, *Ifi27l2a*, *Ifi44*, *Ifi44l*) (**Supplementary Table S11**). GSEA demonstrated activation of biosynthetic and metabolic programs such as cytoplasmic translation, ribosome biogenesis, oxidative phosphorylation (NES = 2.5–3.0, FDR < 0.001), alongside interferon-alpha response (NES = 1.94, FDR = 0.002), with concurrent suppression of immunosuppressive pathways including TNF-α/NF-κB signaling, and IL-6/JAK/STAT3 signaling (NES = −1.9 to −2.4, FDR < 0.05) (**Figure 7B**; **Supplementary Table S12**).

To quantitatively assess the relationship between Asha-induced neutrophils and functionally defined neutrophil states, we calculated module scores for the seven transcriptional hubs mapped in the NeuMap reference atlas (26). Asha Therapy was associated with a significant enrichment of the IFN-response hub signature (median score: Control = 0.21, Asha = 0.46; p = 0.041, **Figure 7C**), a transcriptional state characterized by interferon-stimulated gene expression, metabolic activation, and anti-tumor function. Negligible changes were observed in other hub signatures (**Supplementary Figure S7**; all Cohen’s d < 0.15). Critically, neutrophils showed no enrichment of immunosuppressive (IS-I, IS-II) or immature hub signatures (p > 0.10), indicating selective activation of the IFN-response trajectory rather than non-specific inflammatory reprogramming. This pattern of interferon activation with suppressed classical inflammatory pathways is consistent with a shift away from the immunosuppressive, pro-tumorigenic neutrophil states typically observed in untreated tumors. To further characterize this response, we generated a bespoke Neutrophil Activation Score from the top differentially expressed genes in neutrophils exposed to Asha Therapy to create a human-projectable signature for clinical cohort validation (**Supplementary Material 1**). When applied back to the discovery dataset, this score reflected the expected shift in the treated population (median score: Control = -0.077, Asha = 0.252; p = 0.008; **Figure 7D**).

At the protein level, CITE-seq analysis was consistent with the transcriptomic findings, showing significant upregulation of activation markers including CD9 (log_2_FC = +1.41), CD103 (log_2_FC = +0.89), and CD11c (log_2_FC = +0.78), as well as the interferon-stimulated surface antigen Ly6A/E (Sca-1; log_2_FC = +1.00), with reduced Ly6G (log_2_FC = −0.76) (**Figure 7E**; **Supplementary Table S13**). These transcriptomic and proteomic signatures jointly support the interferon-driven activation phenotype.

These findings demonstrate that Asha Therapy is associated with selective expansion of neutrophils and a shift toward a metabolically activated, interferon-responsive state associated with tumor control in immunotherapy contexts. This interferon-driven phenotype contrasts with polymorphonuclear myeloid-derived suppressor cells (PMN-MDSCs), which suppress anti-tumor immunity through arginase-1, ROS, and T cell inhibition, and whose accumulation correlates with poor outcomes in many cancers.

### 3.12 Projection of the neutrophil ISG program onto human TNBC tumors

To test whether the neutrophil program we observed in mice also appears relevant in human breast cancer, we projected our 19-gene neutrophil ISG signature onto the METABRIC TNBC cohort (n = 348), SCAN-B cohort (n=874), and the TCGA-BRCA TNBC cohort (n=191). In the METABRIC and SCAN-B cohorts, high NSG score was associated with significantly improved overall survival by Kaplan–Meier analysis (**Supplementary Figures S8A, B**, log-rank p=0.036 and p=0.048). In both these cohorts individual multivariable Cox models adjusting for nodal status, T stage, TNBC subtype, and stratifying by age, showed a significant protective effect (METABRIC: HR = 0.67, 95% CI: 0.49-0.92, p=0.01; SCAN-B: HR = 0.73, 95% CI 0.54-0.98, p=0.04; **Figure 8**).

**Figure 8:**
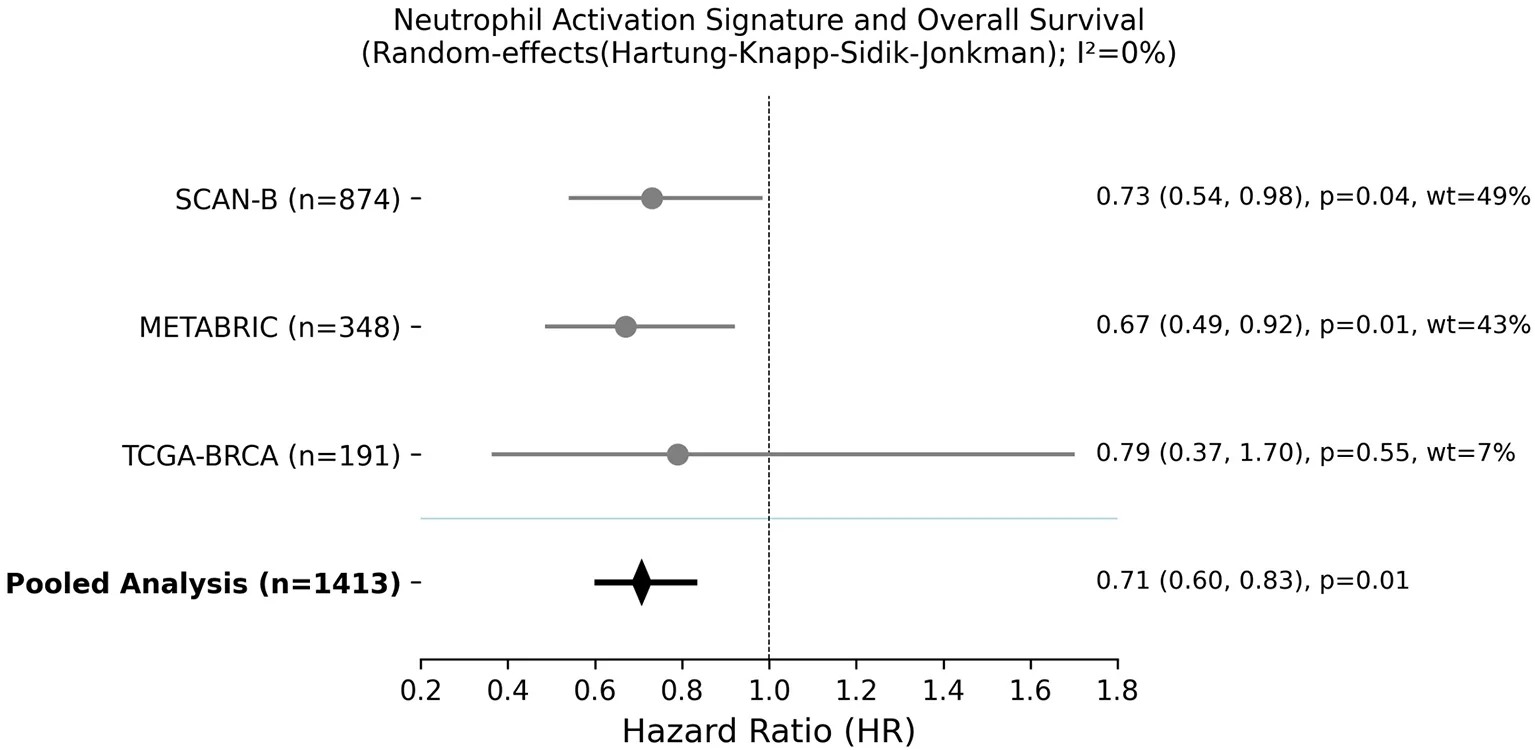
Association of the Neutrophil Activation Signature with overall survival across three independent TNBC cohorts. Hazard ratios (HR) and 95% confidence intervals for overall survival were estimated by multivariable Cox proportional-hazards regression in SCAN-B (n=874), METABRIC (n=348), and TCGA-BRCA (n=191), adjusting for nodal status (N0 vs N1+), tumor stage (T1 vs T2-4), and TNBC subtype (basal vs others), with age included as a stratification factor. Cohort-level estimates were pooled using Hartung-Knapp-Sidik-Jonkman method. Per-cohort percentages (wt) indicate each cohort’s contribution to the pooled estimate. High neutrophil activation score was associated with improved overall survival in all three cohorts (heterogeneity statistics, I²=0%, indicating three cohort results are consistent with each other), reaching independent statistical significance in METABRIC (p=0.01) and SCAN-B (p=0.04). The smaller TCGA-BRCA cohort showed the same protective direction but did not reach significance. The pooled estimate was statistically significant (n=1,413 total; pooled HR = 0.71, 95% CI 0.60–0.83, p=0.01). The dashed vertical line indicates HR = 1 (no association).

In the smaller TCGA-BRCA cohort, high NSG score was not significantly associated with improved survival (**Supplementary Figure S8C**, log-rank p=0.47), and a multivariable Cox model adjusted for nodal status, T stage, TNBC subtype, and stratifying by age, showed a protective effect but did not reach significance (HR = 0.79, 95% CI: 0.37-1.70, p=0.55; **Figure 8**). Given the limited number of events in TCGA-BRCA (n=30), this subtype-adjusted estimate should be interpreted with caution relative to the larger METABRIC and SCAN-B cohorts.

When all three cohorts were pooled together using a random-effects model, the protective association was statistically significant (pooled HR = 0.71, 95% CI 0.60–0.83, p=0.01; **Figure 8**), with no detected heterogeneity between cohorts (I²=0%), indicating a reproducible survival benefit of high NSG score across datasets.

The prognostic value of the neutrophil activation signature is notable given that it was derived from high-resolution single-cell neutrophil profiling of Asha-treated murine tumors and projected onto bulk RNA from human TNBC biopsies. Despite the cross-species translation and inherent cellular heterogeneity of bulk RNA, the signature maintained a significant association with survival across the two large independent cohorts and was protective, though non-significant, in the smaller sized cohort. This conservation across species and data modalities suggests the identified neutrophil activation program has intrinsic prognostic relevance in TNBC.

### 3.13 Asha Therapy is associated with interferon-responsive transcriptional and surface marker changes in tumor-associated monocytes and macrophages

Macrophages and monocytes are key regulators of tumor immunity and can exhibit either pro-tumor or anti-tumor functions. We conducted exploratory analysis to examine Asha Therapy effects on these populations. Re-clustering identified four mononuclear phagocyte subsets: C1qa^+^Trem2^+^Apoe^+^ lipid-associated macrophages (LAMs, 48.8%), Ly6c2^+^Ccr2^+^ inflammatory monocytes (25.6%), Mrc1^+^Arg1^+^ IL4-responsive macrophages (21.7%), and MHC-II^+^ macrophages (3.8%) (**Supplementary Figures S9A–C**). Compositional analysis (scCODA) showed stable subset proportions (**Supplementary Figure S9D**).

LAMs, the dominant population (48.8%), showed enrichment of type I interferon response (IFN-α, NES = 2.18; IFN-γ, NES = 2.03), antimicrobial defense pathways (NES = 2.29), and neutrophil migration signatures (NES = 1.96), alongside suppressed oxidative phosphorylation (NES = -2.28) and MYC targets (NES = -2.17) (**Figure 9A**; **Supplementary Tables S14**, **S15**). At the protein level, CITE-seq showed increased expression of the interferon-inducible protein Sca-1 (log_2_FC = +0.79) but reduced antigen-presentation markers MHC-II (log_2_FC = -0.57) and CD86 (-0.39) (**Figure 9B**; **Supplementary Table S16**).

**Figure 9:**
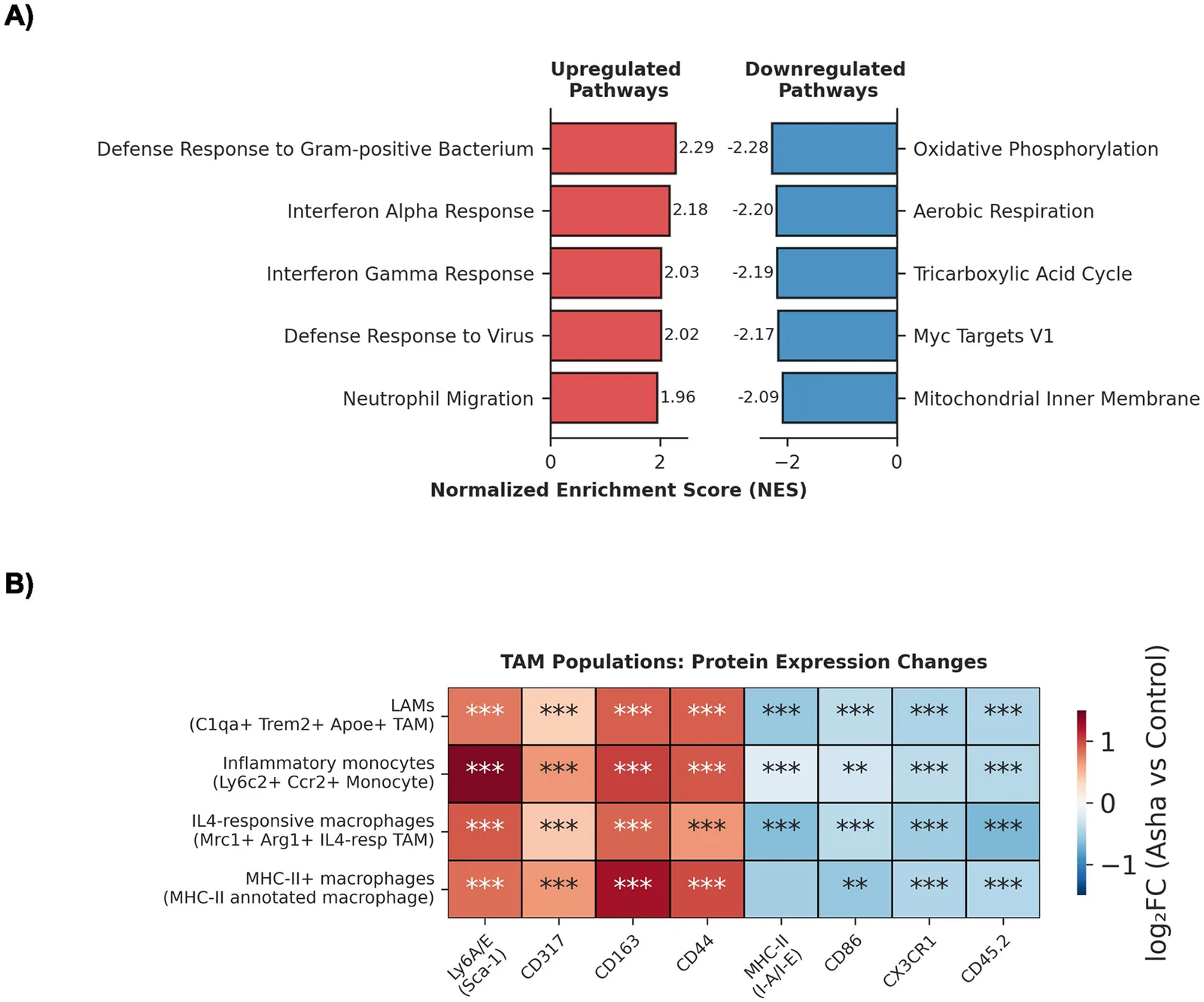
Asha therapy is associated with transcriptional and surface-marker shifts within tumor-associated macrophage/monocyte subsets. **(A)** Gene Set Enrichment Analysis (GSEA) of the C1qa^+^Trem2^+^Apoe^+^ lipid-associated macrophage (LAM) subset following therapy. Bar plots show normalized enrichment scores (NES) for significantly altered pathways (FDR < 0.05). Asha therapy is associated with upregulation of interferon-alpha/gamma response and antimicrobial defense pathways alongside neutrophil migration signatures, and downregulation of metabolic/biosynthetic programs including oxidative phosphorylation, aerobic respiration, and Myc targets. **(B)** Differential surface protein expression across myeloid subsets. Heatmap shows log_2_(fold-change) (Asha vs. Control) for TAM subsets: C1qa^+^Trem2^+^Apoe^+^ lipid-associated macrophages (LAMs), Ly6c2^+^Ccr2^+^ inflammatory monocytes, Mrc1^+^Arg1^+^ IL4-responsive macrophages, and MHC-II^+^ macrophages. Red indicates upregulation, blue indicates downregulation. The interferon-responsive marker Ly6A/E (Sca-1) and CD317 were upregulated across the profiled subsets, alongside downregulation of MHC-II and CD86. These shifts reflect surface-phenotype and transcriptional changes within existing myeloid populations rather than a compositional shift between subsets (see **Supplementary Figure S7**). Significance was determined via Wilcoxon rank-sum test with Benjamini-Hochberg correction; ***, **, and * correspond to p < 0.001, p < 0.01, and p < 0.05, respectively.

IL4-responsive macrophages showed similar changes (increased Sca-1, +0.91; reduced MHC-II, -0.64; CX3CR1, -0.53) with enrichment of alarmin-driven pathways (S100a8, S100a9; NES = 2.39) (**Figure 9B**; **Supplementary Tables S14**-**S16**). Inflammatory monocytes and MHC-II^+^ macrophages exhibited protein-level interferon signatures (increased Sca-1, CD317; reduced CX3CR1) with minimal transcriptional changes (**Figure 9B**; **Supplementary Tables S14**-**S16**).

### 3.14 Asha Therapy is associated with biosynthesis and metabolic transcriptional and surface marker changes in the T cell compartment

We conducted an exploratory analysis to evaluate transcriptional and surface marker changes in T cells within the tumor microenvironment following Asha Therapy. Re-clustering of the T-cell compartment identified five transcriptionally distinct subsets: exhausted CD8^+^ (29.1%), regulatory T cells (26.3%), CD4^+^ helper (18.3%), CD8^+^ memory-like (13.0%), and γδ T cells (13.4%) (**Supplementary Figures S10A–C**). Compositional analysis (scCODA) showed stable subset proportions (**Supplementary Figure S10D**).

All αβ T-cell populations showed biosynthetic pathway enrichment (cytoplasmic translation and ribosome biogenesis, NES = 2.1–3.2, FDR < 0.001). Pathway-level differences were observed across subsets. Exhausted CD8^+^ T cells showed enrichment of TNF-α/NF-κB signaling (NES = 2.33) and upregulation of Ifng (log_2_FC = +1.82), alongside increased surface activation markers (CD3, CD44, Sca-1) (**Supplementary Figure S11A**; **Supplementary Tables S17**-**S19**). In contrast, CD4^+^ helper and CD8^+^ memory-like cells exhibited ribosomal enrichment (NES = 2.7–3.2) but suppressed TCR signaling (NES = -2.2 to -2.3) and reduced co-stimulatory markers (GITR, ICOS, TCRβ) (**Supplementary Figure S11A**; **Supplementary Tables S17**-**S19**).

Regulatory T cells showed biosynthetic and inflammatory activation (TNF-α/NF-κB, NES = 2.05; IFN-α response, NES = 2.00) alongside reduced expression of suppressive surface markers GITR (log_2_FC = -1.27), ICOS (-0.97), and CD103 (-0.90) (**Supplementary Figure 11A**; **Supplementary Tables S17**-**S19**). γδ T cells displayed interferon-driven pathway enrichment (IFN-α response, NES = 2.44) without ribosomal activation, with reductions in tissue residency markers (integrin β7, -1.37; CD103, -1.20; TCR Vγ2, -1.06) and co-stimulatory molecules (GITR, -1.68; ICOS, -1.35).

## 4 Discussion

Using multi-omics analysis across *in vitro* models and *in vivo* tumor microenvironments, we demonstrate that low-intensity EMF-based Asha Therapy is associated with coordinated tumor and stromal transcriptional and surface marker changes correlated with reduced metastatic progression, and enhanced responsiveness to the immune checkpoint blockade. Importantly, we identify a selective shift of neutrophils toward an interferon-activated anti-tumor state as a key component of the immune response associated with Asha Therapy.

### 4.1 Asha Therapy shifts malignant cells to an immunogenic stress state

A key tumor-intrinsic response associated with Asha Therapy is the activation of adaptive ER stress without inducing acute cytotoxicity. This stress response is not static, it matures from an initial disruption of protein folding accompanied by secretion of immune-recruiting factors. Within 48–96 hours, tumor cells show a reciprocal downregulation of anabolic mTOR effectors and an upregulation of the stress-adaptive cAMP-PKA pathway. The induction of ITPKA is consistent with a role for calcium signaling in this stress response, though the biophysical link between electromagnetic exposure and this pathway has not been directly tested.

This stress program evolves under chronic *in vivo* exposure. While acute *in vitro* responses are dominated by NF-kB activation, malignant cells after seven days of continuous treatment exhibit a transition toward stable, interferon-driven states with suppressed NF-κB signaling. The temporal dynamics of this NF-κB response, activation during acute stress followed by suppression under chronic exposure, are consistent with adaptation from pro-inflammatory to interferon-dominant signaling, and an active metabolic and biosynthetic response. These cells showed high metabolic activity, increased Sca-1 expression, and reduced expression of immunosuppressive markers like CD73 and the stemness-associated factors CD49f and CD24. These findings are consistent with Asha Therapy being associated with a sustained shift in tumor cell state that may increase susceptibility to immune surveillance, though this has not been directly tested functionally.

We note that the observed inflammatory secretory program shares features with a senescence-associated secretory phenotype (SASP)-like or broader adaptive stress response, and our data cannot fully distinguish an ER-stress-specific secretory program from a more general adaptive stress response of which ER stress is one component. The temporal pattern we observed, an early, transient induction that resolves by 72–96 hours, is more consistent with an acute adaptive stress response than with a stable, typically sustained senescence-associated phenotype, but we present this as an open question rather than evidence for an exclusively ER-stress-driven mechanism.

### 4.2 Selective neutrophil reprogramming as a key feature of the Asha Therapy response

Among all immune populations in the TME, neutrophils exhibited the most pronounced response to Asha Therapy, characterized by both a significant compositional expansion and a transcriptional and surface marker shift. This pattern is consistent with recently described interferon-activated anti-tumor states (24, 26). By mapping our data against established neutrophil atlases, we noticed a selective activation of these anti-tumor programs rather than a nonspecific inflammatory response. These findings identify neutrophil transcriptional and surface marker activation as a prominent feature of the Asha Therapy-associated immune response.

The clinical relevance of this neutrophil program is supported by its association with improved outcomes in human TNBC. A 19-gene activation signature, derived directly from Asha-treated murine neutrophils identified a significant pooled association with improved overall survival across three independent cohorts (pooled HR = 0.71, 95% CI 0.60–0.83, p=0.01, adjusted for T-stage, nodal status, TNBC intrinsic subtype, and stratified by age), reaching individual significance in two of the three (METABRIC and SCAN-B). This cross-species conservation, where a murine treatment-induced signature is prognostic in human outcomes, despite the heterogeneity of bulk-RNA biopsies, supports the biological relevance of the Asha-induced neutrophil signature. These results suggest that targeting or monitoring this specific neutrophil program could offer a new therapeutic or biomarker-driven roadmap for managing aggressive breast cancers. We emphasize that these findings establish the neutrophil activation signature as a prognostic signal supported by cross-cohort association, not as a validated clinical biomarker. Formal prospective clinical validation remains a necessary next step before any diagnostic or therapeutic application. We further note that the signature overlaps biologically with interferon-related programs. In bulk human tumor data, where cell-type resolution is lost, we cannot yet confirm the signature specifically reflects neutrophil activation rather than a broader interferon response.

The biological plausibility of this proposed sequence does not rest on a single correlative observation, but on three lines of evidence that converge in a temporally ordered pattern. First, core ER stress/UPR markers (GRP78, CHOP, ATF4, XBP1s) were induced within 24–48 hours of Asha exposure at both the transcriptomic level and via single-cell flow cytometry protein quantification. Second, an immune-recruiting secretory program (IL-6 and ADAMTS1, followed temporally by CXCL10, CCL2, CCL20, and CXCL1) emerged at matched and subsequent timepoints across secretomic and transcriptomic platforms. Third, neutrophil expansion and interferon-pathway activation were captured *in vivo* within the myeloid/immune compartment of Asha-treated tumors by CITE-seq. While this does not constitute definitive causal proof, it represents a temporally consistent signal observed across four independent technical platforms (bulk RNA-seq, global proteomics, targeted secretomics, and CITE-seq) and two distinct experimental systems (*in vitro* human TNBC lines and *in vivo* syngeneic murine tumors). This plausibility is further supported by recent literature describing therapy-induced anti-tumor neutrophil activation states in other contexts within which our findings align (24, 25).

### 4.3 Parallel transcriptional and surface marker changes across stromal, myeloid, and T-cell compartments

Asha Therapy is associated with changes in the TME **t**hrough both compositional and functional changes in myeloid populations. Neutrophils expanded significantly, while macrophages and monocytes underwent transcriptional and surface marker changes, without changes in relative abundance. These transcriptional patterns are consistent with an interferon-associated shift across the stromal and myeloid compartments, though this remains a hypothesis-generating observation given the single-timepoint, n=3 design of the CITE-seq dataset. CAFs showed reduced expression of markers associated with contractile, matrix-depositing programs, which typically facilitate metastasis, alongside increased expression of markers associated with stress-responsive phenotypes with a potential for neutrophil-recruitment capacity. Simultaneously, tumor-associated macrophages showed an innate inflammatory transcriptional profile, including interferon-inducible stress responses despite reduced antigen-presentation markers (MHC-II, CD86).

These findings raise the possibility of a “priming” effect on adaptive immunity, though this has not been directly tested. We observed widespread biosynthetic and metabolic pathway enrichment across the T-cell compartment, consistent with CD4+ and memory-like CD8+ T-cells showing pathway-level changes despite a lack of effector marker changes. Furthermore, a reduction in suppressive markers on regulatory T-cells is consistent with a localized weakening of immune suppression. These transcriptional and marker-level findings across the TME provide a plausible, though unproven, mechanistic context for the significantly reduced lung metastasis and 88% reduction in death risk (HR = 0.12) observed when Asha Therapy is combined with anti-PD1. By potentially shifting a contractile, immunosuppressive stroma toward an interferon-rich, metabolically active environment, Asha Therapy may enhance responsiveness to checkpoint blockade specifically in metastatic disease. Notably, these effects occurred without significant primary tumor growth inhibition, suggesting that Asha-induced immune changes may be effective in the metastatic niche.

### 4.4 Asha Therapy represents a potential non-cytotoxic paradigm for EMF-based oncology treatment

The proposed mechanism of Asha Therapy differs from existing physical tumor interventions. While Tumor Treating Fields (TTFields) achieve anti-tumor effects through mitotic disruption at higher frequencies (74), and oncomagnetic therapies focus on acute mitochondrial ROS production (34), Asha’s low-intensity fields are associated with an adaptive stress and altered tumor cell states rather than the direct cytotoxicity characteristic of these other modalities.

This mechanism has direct translational implications for overcoming primary checkpoint resistance in triple-negative breast cancer (TNBC). The improved response to anti-PD1 therapy observed here, in a model with limited monotherapy efficacy, may help address an unmet need for patients who derive limited benefit from current checkpoint inhibitors. The neutrophil-centric transcriptional and surface marker changes identified here offer a potential roadmap for clinical monitoring: interferon-stimulated gene signatures in peripheral blood or biopsies could serve as potential pharmacodynamic biomarkers to guide therapy. While continuous administration in mice was well-tolerated without overt toxicity, formal dose-ranging and toxicity studies in normal tissues will be essential for clinical translation. Taken together these findings suggest Asha Therapy as a candidate non-pharmacologic strategy to improve responsiveness of aggressive tumors to immunotherapy.

### 4.5 Limitations and future directions

While our findings establish a proof-of-concept framework for Asha Therapy, several limitations should be acknowledged. The proposed mechanistic sequence linking ER stress, neutrophil reprogramming, and therapeutic efficacy is supported by temporally ordered, cross-platform correlative evidence, but has not been established through direct perturbation. Neutrophil depletion (e.g., anti-Ly6G) would be required to establish that neutrophils are necessary for the observed therapeutic benefit, and pharmacological UPR inhibition would be required to establish a causal role for ER stress in the downstream immune-recruiting response; both represent priorities for follow-up studies. The precise biophysical link between low-intensity EMF exposure and ER stress requires further validation through live cell imaging and genetic perturbation of identified hubs like ITPKA. Similarly, the temporal transition from acute NF-κB activation to a sustained interferon state likely involves intermediate regulatory steps that, if identified, could be exploited to optimize dosing protocols.

Our efficacy studies were restricted to the 4T1 murine model and combination with anti-PD1 blockade. Critically, whether the observed survival benefit is dependent on an intact immune system remains to be formally tested in immune-deficient models. *in vitro* characterization was performed across multiple TNBC cell lines; while this reflects practical considerations, cross-validation of key findings across a single standardized model would strengthen mechanistic conclusions. CITE-seq profiling was performed at n=3 per group, consistent with published tumor microenvironment studies, though findings should be interpreted in the context of this sample size. Convergent evidence from bulk RNA-seq and proteomics supports the transcriptional stress and secretory program findings, the human cohort survival association supports the prognostic relevance of the derived gene signature but does not independently validate the specific single-cell compositional or phenotypic interpretations, which remain dependent on the CITE-seq dataset itself pending orthogonal validation. Additionally, individual cohort Cox models showed a consistent protective direction across all three cohorts, reaching significance in METABRIC and SCAN-B; TCGA-BRCA showed the same direction but did not reach significance, consistent with its smaller number of events (n=30), and subtype-adjusted estimates in this cohort should be interpreted with caution given the limited event count. Finally, Asha Therapy effects on normal stromal and immune cells in healthy tissue remain uncharacterized, and formal dose-ranging and toxicity studies will be essential for clinical translation. Formal measurement of physiological stress markers, such as serum corticosterone or behavioral stress indices, was not performed in this study and would help formally exclude a generalized stress response as a contributor to the observed effects; we consider this a priority for future studies using this device.

Exploring combination benefit with standard-of-care chemotherapy or alternative immunotherapies, such as CTLA-4 blockade, remains a critical translational priority. Finally, systematic optimization of field parameters (including frequency, intensity, and duty cycle) will be essential to optimize the TME response in human patients. Addressing these areas will be vital to moving EMF-based immune priming from a preclinical discovery toward a viable clinical intervention for aggressive cancers.

## 5 Conclusions

This work provides a preclinical rationale for further investigation of Asha Therapy as a strategy to modulate the TME in TNBC. Asha Therapy’s low-intensity EMF was associated with adaptive ER stress in tumor cells, alongside TME changes including selective neutrophil expansion and interferon-driven transcriptional and surface marker changes. Additional transcriptional and surface marker changes were observed across monocyte/macrophage, fibroblast, and T cell compartments, which we present as hypothesis-generating given the exploratory nature of this single-timepoint dataset. Combined with checkpoint blockade, these changes were associated with therapeutic benefit in this preclinical model, supporting further investigation of Asha Therapy as an immune priming strategy in TNBC.

The temporal pattern from acute stress to a shifted TME, and the neutrophil-centric findings described here, represent a preclinical foundation for further study of how EMF-based modalities may influence tumor microenvironments. These findings support Asha Therapy as a candidate immunomodulatory strategy that warrants further investigation for its potential to enhance checkpoint responsiveness in TNBC.

## Data Availability

The data presented in the study are deposited in the GEO repository, accession number GSE350789.
