# Supplementary material for: Non-invasive electromagnetic fields induce anti-tumor neutrophil reprogramming and enhance checkpoint blockade in breast cancer
Source: Front Immunol. 2026 Oct 16;17:1861048. doi: 10.3389/fimmu.2026.1861048 (PMC13720674; doi:10.3389/fimmu.2026.1861048)
Supplement: Supplementary file 2 [file DataSheet10.pdf]

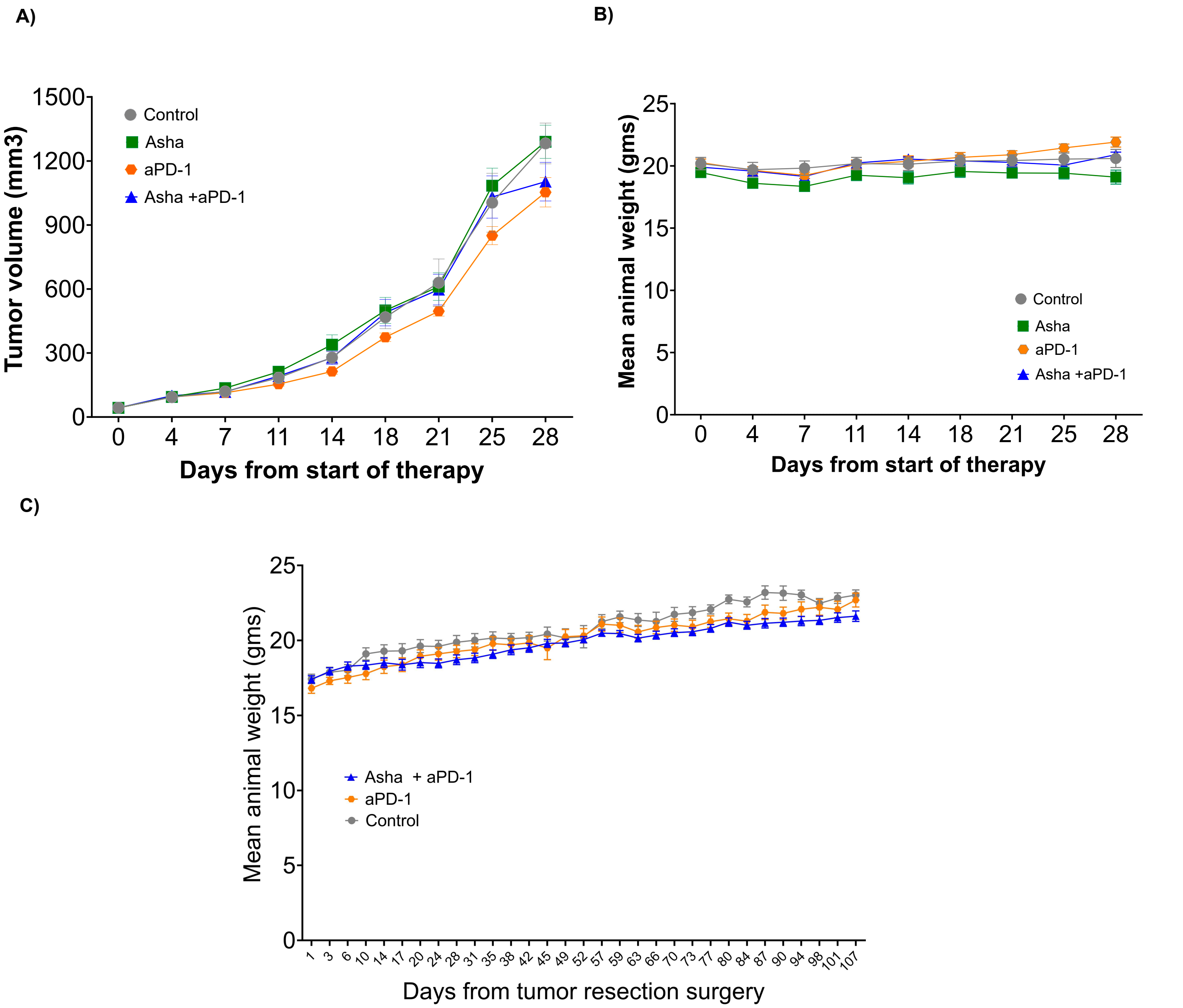

Supplementary Figure S4: A) Longitudinal measurement of primary tumor volume (mm^3) in the 4T1 orthotopic model across experimental groups (Control, Asha, anti-PD1, and Combination) in the 28-day metastasis study (n=6/group). Data represent mean ± SEM. No significant differences in primary tumor volume were observed across groups at the time of surgical resection or study completion. B) Body weight measurements over the 28-day treatment period for mice in the metastasis study. Data represent mean ± SEM. The stability of body weight across all groups indicates that Asha therapy and combination regimens were well-tolerated. C) Body weight measurements over the survival study after primary tumor was resected. Data represent mean ± SEM. The absence of weight loss throughout the monitoring period in both studies indicates that therapy has a good safety profile.
