# Supplementary material for: Non-invasive electromagnetic fields induce anti-tumor neutrophil reprogramming and enhance checkpoint blockade in breast cancer
Source: Front Immunol. 2026 Oct 16;17:1861048. doi: 10.3389/fimmu.2026.1861048 (PMC13720674; doi:10.3389/fimmu.2026.1861048)
Supplement: Supplementary file 3 [file DataSheet11.pdf]

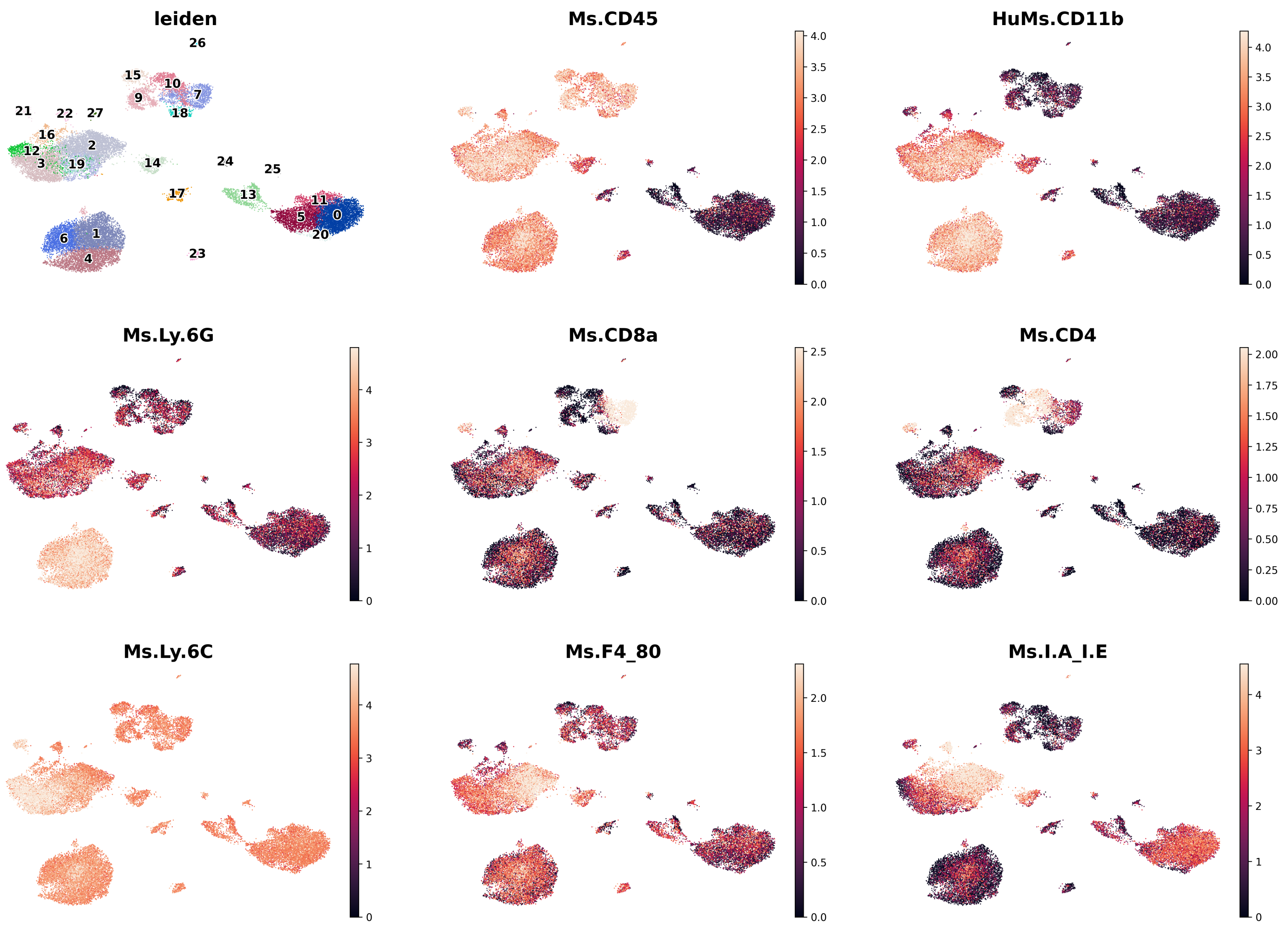


Supplementary Figure S5: Leiden cluster annotations validated by CITE-seq surface protein expression (CD45, CD11b, Ly6G, CD8a, CD4, Ly6C, F4/80, IA/IE)
