# Supplementary material for: Non-invasive electromagnetic fields induce anti-tumor neutrophil reprogramming and enhance checkpoint blockade in breast cancer
Source: Front Immunol. 2026 Oct 16;17:1861048. doi: 10.3389/fimmu.2026.1861048 (PMC13720674; doi:10.3389/fimmu.2026.1861048)
Supplement: Supplementary file 4 [file DataSheet2.pdf]

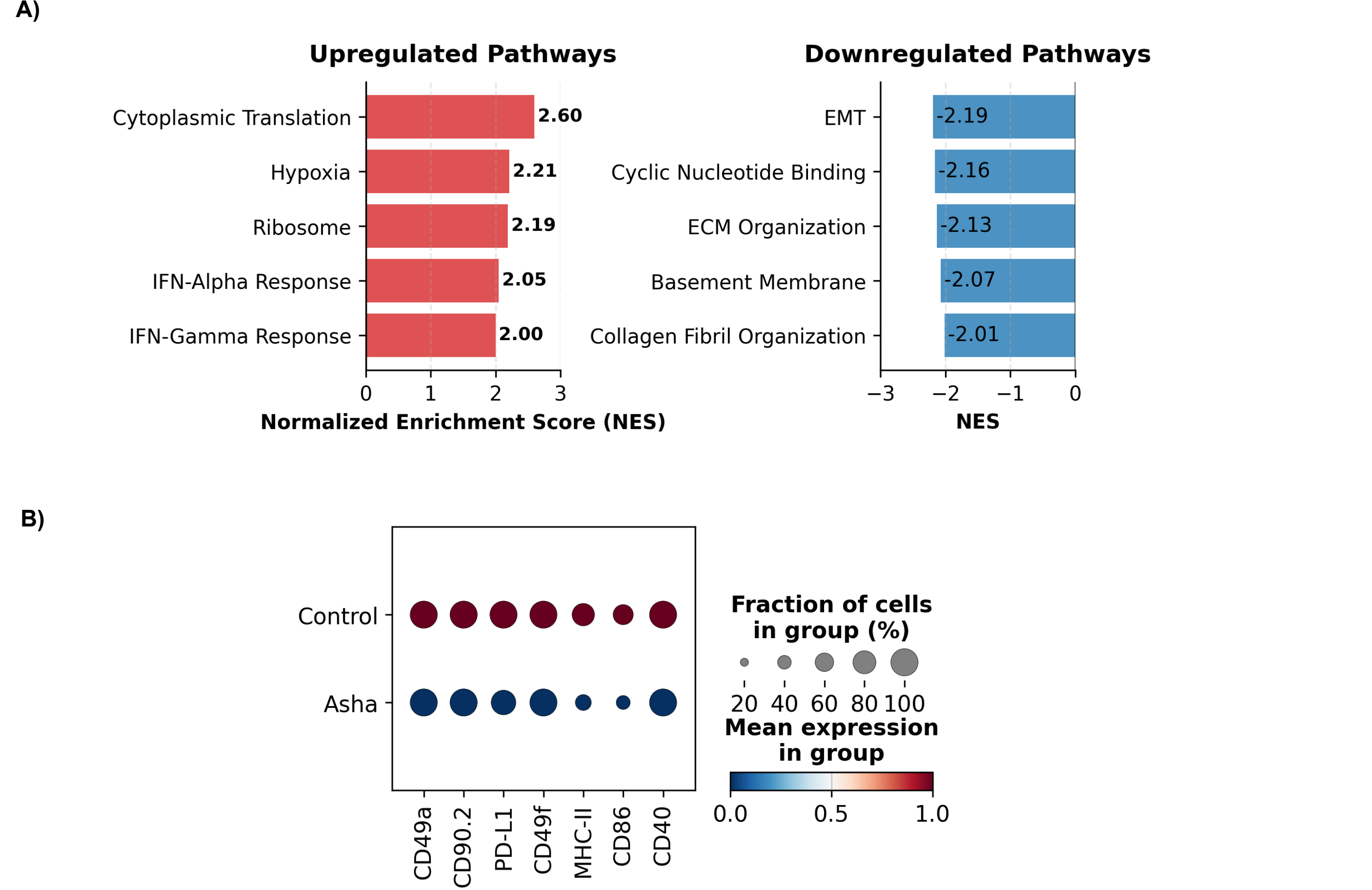

Supplementary Figure S6: (A) Gene Set Enrichment Analysis (GSEA) of fibroblasts comparing Asha-treated vs. Control tumors. Bar plot displays representative biological programs significantly enriched (FDR < 0.05), ranked by Normalized Enrichment Score (NES). Upregulated pathways (red) indicate a metabolic reprogramming toward a biosynthetically active (e.g., Cytoplasmic Translation) and interferon-responsive phenotype. Downregulated pathways (blue) indicate a suppression of contractile (EMT) and matrix-associated programs (ECM Organization). (B) Dot plot displaying CITE-seq protein expression for key structural and immunomodulatory markers in the fibroblasts. Markers include the myofibroblast marker Thy1 (CD90.2), integrin subunits (CD49a, CD61), and the immune-checkpoint ligand PD-L1 (CD274). Color indicates standardized mean expression per marker, and dot size represents the percentage of cells expressing each marker.
