# Supplementary material for: Non-invasive electromagnetic fields induce anti-tumor neutrophil reprogramming and enhance checkpoint blockade in breast cancer
Source: Front Immunol. 2026 Oct 16;17:1861048. doi: 10.3389/fimmu.2026.1861048 (PMC13720674; doi:10.3389/fimmu.2026.1861048)
Supplement: Supplementary file 5 [file DataSheet3.pdf]

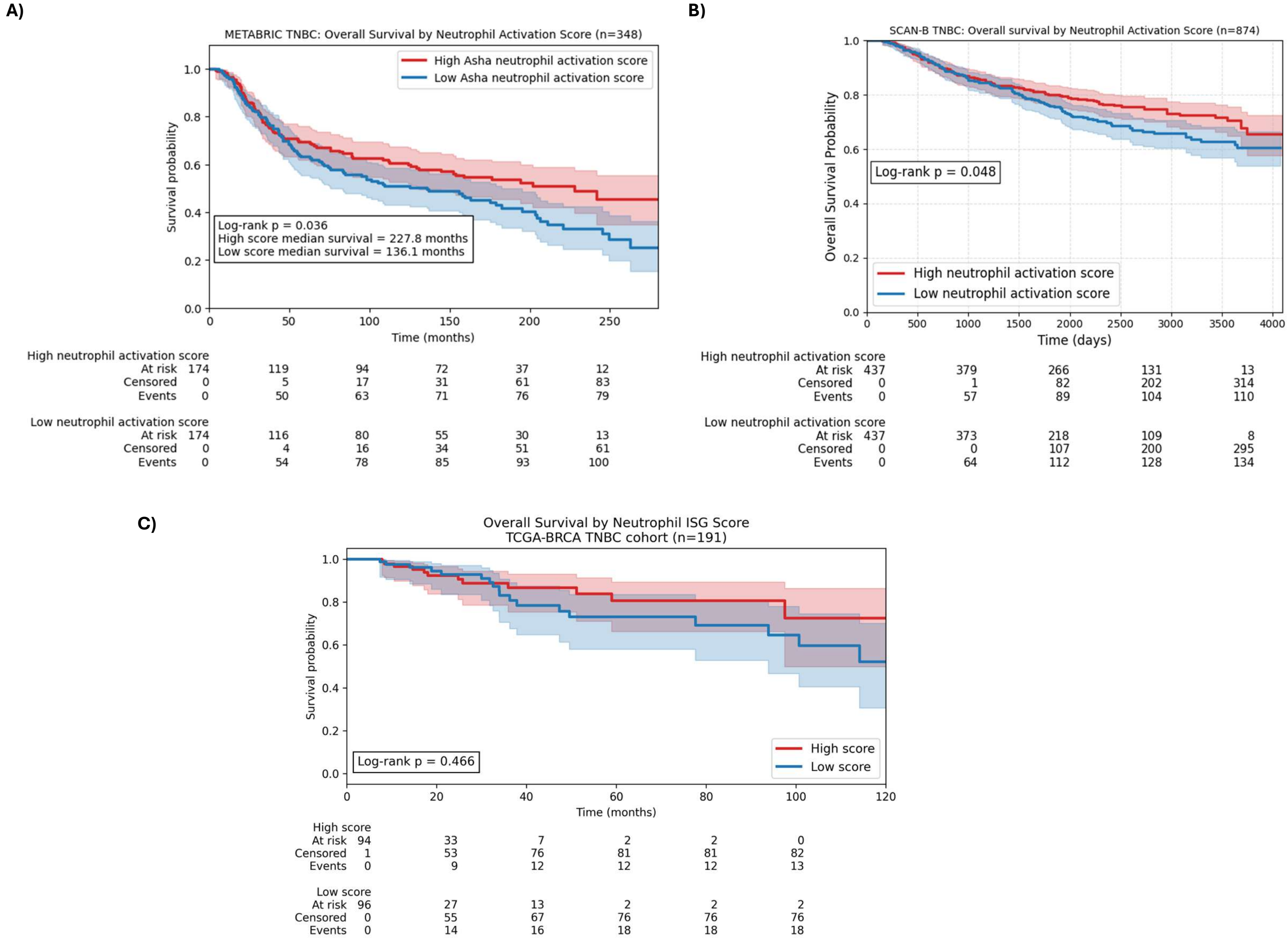

Supplementary Figure S8. Kaplan-Meier survival analysis in three publicly available TNBC cohorts. TNBC patients in each cohort were stratified by the mean expression of the Neutrophil Activation Signature. A) METABRIC cohort (n=348): High signature expression is significantly associated with improved overall survival (Log-rank p=0.036). (B) SCAN-B (n=874): High signature expression is significantly associated with improved overall survival (Log-rank p=0.048). (C) TCGA-BRCA (n=191): High signature expression is not associated with improved overall survival (Log-rank p=0.466). In Kaplan-Meier curves, solid lines represent the survival probability, and shaded areas indicate the 95% confidence intervals.
