# Supplementary material for: Non-invasive electromagnetic fields induce anti-tumor neutrophil reprogramming and enhance checkpoint blockade in breast cancer
Source: Front Immunol. 2026 Oct 16;17:1861048. doi: 10.3389/fimmu.2026.1861048 (PMC13720674; doi:10.3389/fimmu.2026.1861048)
Supplement: Supplementary file 7 [file DataSheet5.pdf]

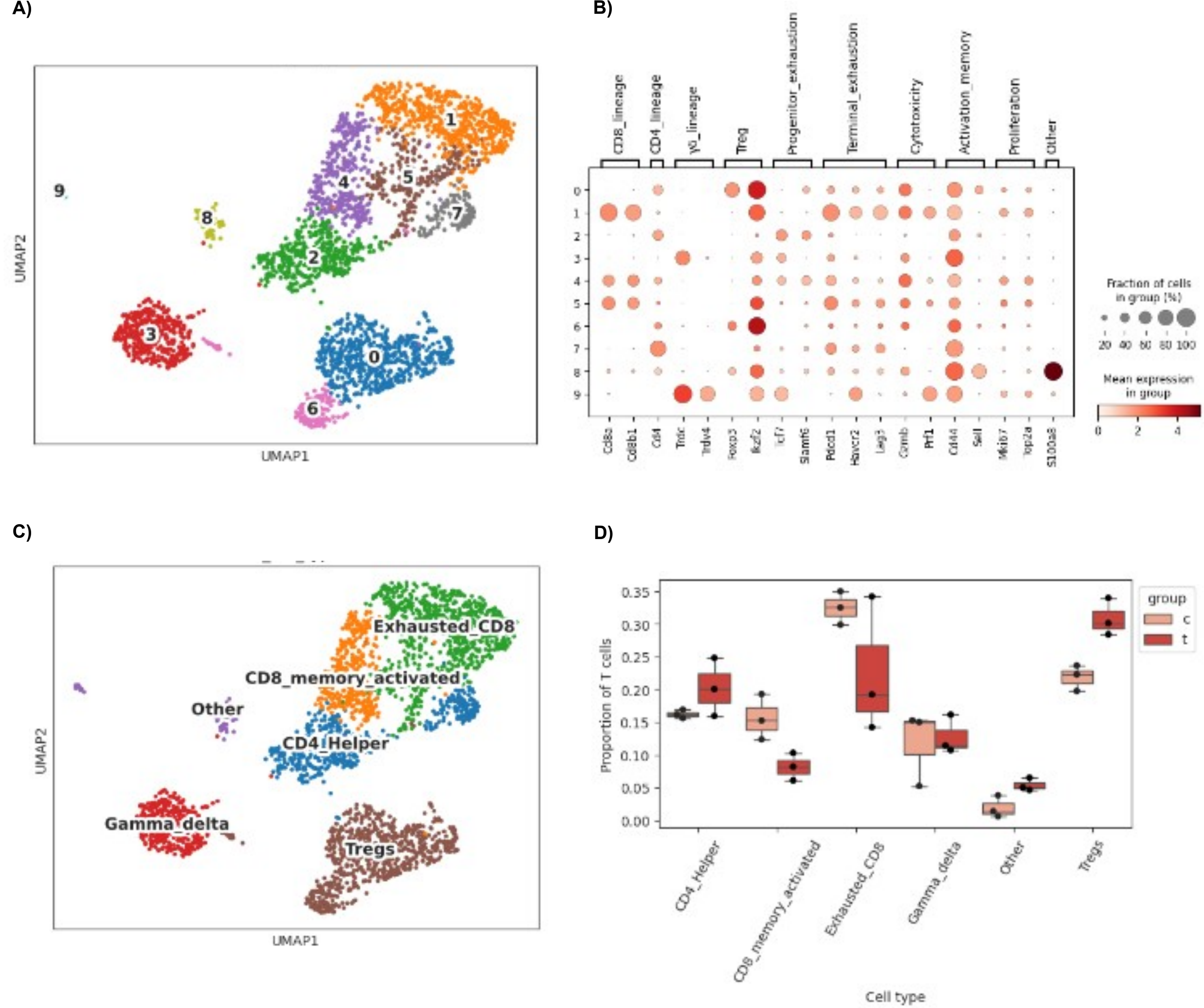

Supplementary Figure S10: Sub-clustering and compositional analysis of the T-cell compartment. A) UMAP visualization of T-cells following Leiden sub-clustering, which identified 10 distinct transcriptional sub-clusters. B) Dot plot illustrating the mean expression and fraction of cells expressing lineage-defining markers across the 10 Leiden clusters. These markers were used to aggregate clusters into five functionally defined cell types: Exhausted CD8+ T-cells (Exhausted_CD8; clusters 1 and 5), regulatory T-cells (Tregs; clusters 0 and 6), CD4+ helper T-cells (CD4_Helper; clusters 2 and 7), CD8+ memory-like (CD8_memory_activated, cluster 4), and gamma-delta T-cells (Gamma_delta; clusters 3). C) UMAP projection labeled by the five aggregated transcriptional subsets used for downstream differential expression and pathway analysis. D) Box plots representing the proportion of identified subsets within the T-cell compartment for Control (c) and Asha-treated (t) groups. Compositional analysis using scCODA confirmed that Asha therapy did not induce significant shifts in the relative proportions of these T-cell populations ($p > 0.05$).
