# Supplementary material for: Non-invasive electromagnetic fields induce anti-tumor neutrophil reprogramming and enhance checkpoint blockade in breast cancer
Source: Front Immunol. 2026 Oct 16;17:1861048. doi: 10.3389/fimmu.2026.1861048 (PMC13720674; doi:10.3389/fimmu.2026.1861048)
Supplement: Supplementary file 8 [file DataSheet6.pdf]

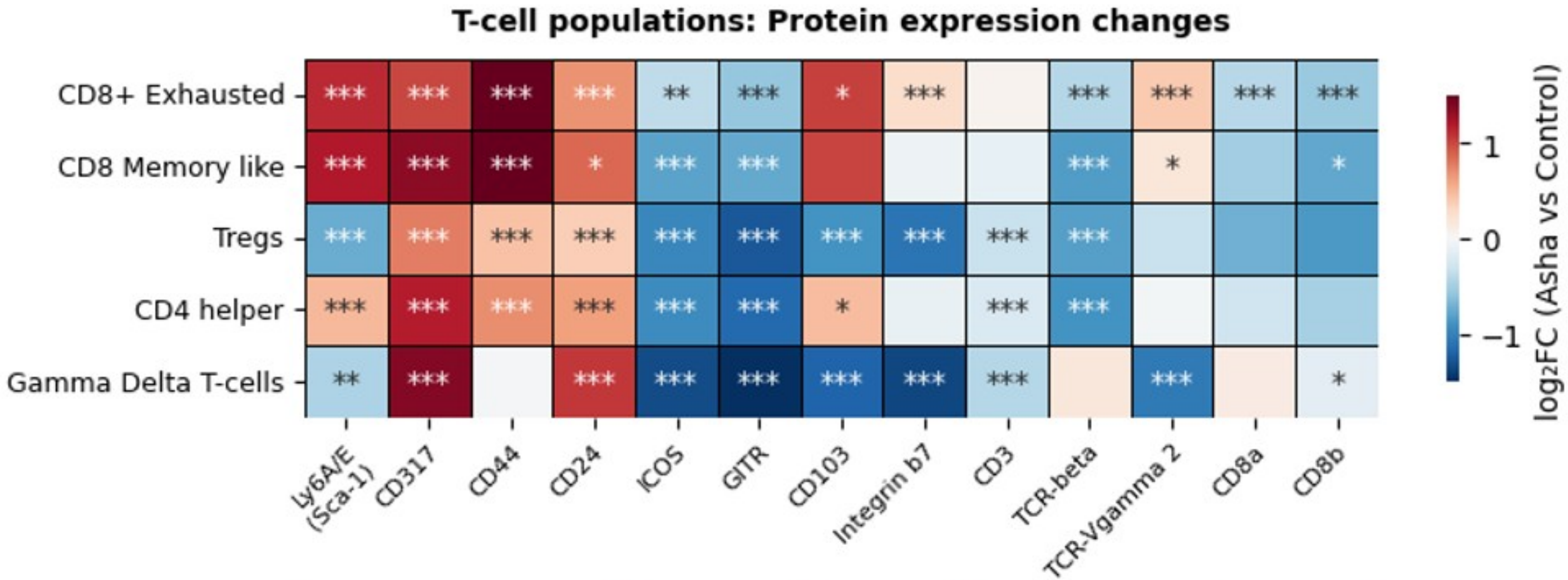

Supplementary Figure S11: Differential surface protein expression across T-cell subsetsHeatmap shows $\log_2$ (fold-change) (Asha vs Control) for T cell subsets: exhausted $CD8^+$ T cells, regulatory T cells (Tregs), $CD4^+$ helper T cells, $CD8^+$ memory-like T cells, and gamma delta T cells. Treatment was associated with upregulation of the interferon-inducible marker CD317 and downregulation of co-stimulatory molecules (GITR, ICOS, CD52) and TCR-beta across all populations. Sca-1 (Ly6A/E) increased selectively in $CD8^+$ and $CD4^+$ subsets but decreased in regulatory T cells. Exhausted $CD8^+$ cells showed increased activation markers (CD3, CD44) with reduced CD8a/b expression, regulatory T cells lost canonical suppressive markers (GITR, ICOS, CD103), and Gamma Delta T cells exhibited reduction in tissue residency markers (integrin β7, CD103) and TCR Vgamma2. Significance was determined via Wilcoxon rank-sum test with Benjamini-Hochberg correction in the heatmap. ***, **, and * correspond to $p < 0.001$, $p < 0.01$, and $p < 0.05$, respectively.
