# Supplementary material for: Non-invasive electromagnetic fields induce anti-tumor neutrophil reprogramming and enhance checkpoint blockade in breast cancer
Source: Front Immunol. 2026 Oct 16;17:1861048. doi: 10.3389/fimmu.2026.1861048 (PMC13720674; doi:10.3389/fimmu.2026.1861048)
Supplement: Supplementary file 9 [file DataSheet7.pdf]

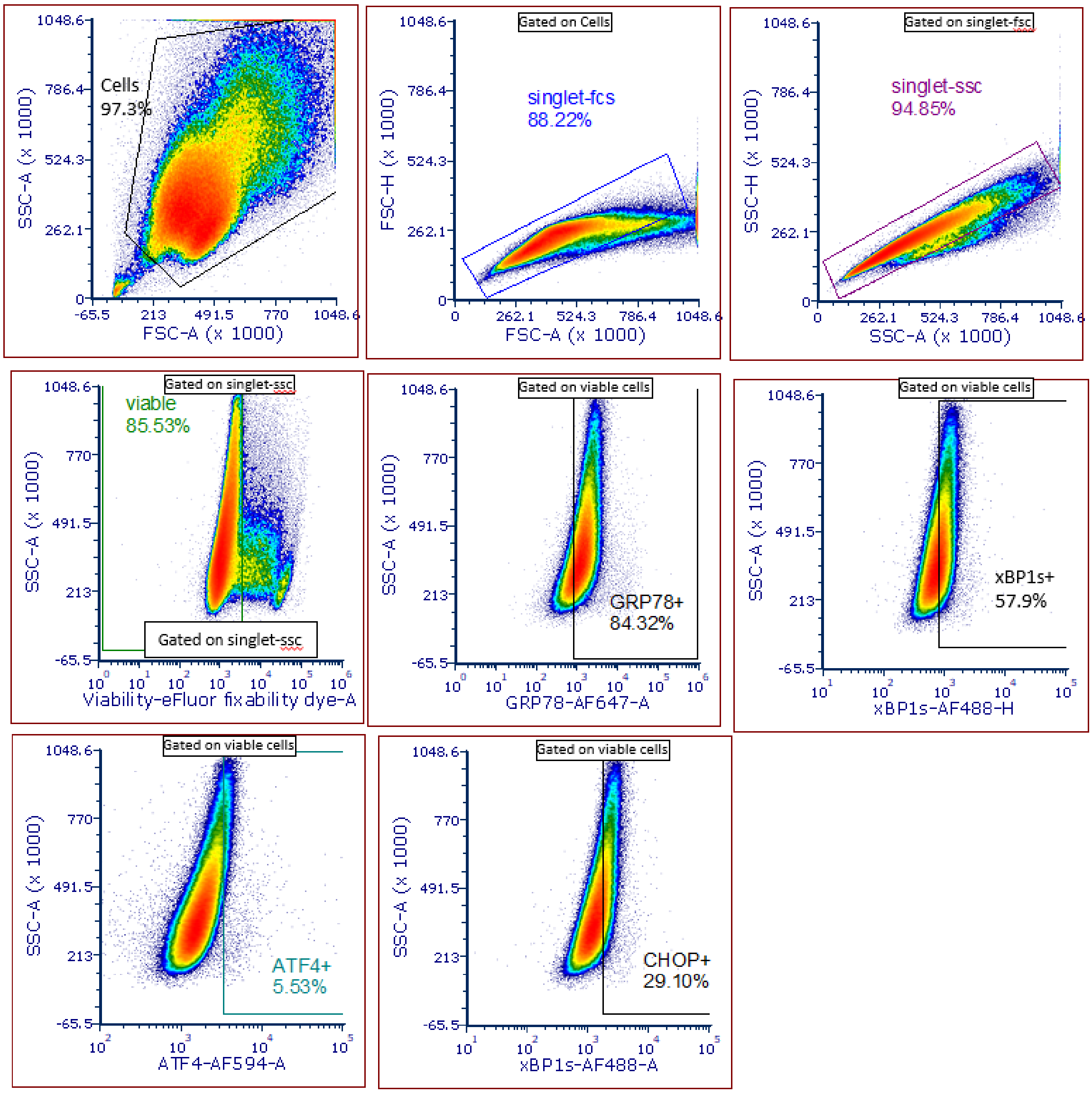

Supplementary Figure S1: Flow cytometer gating used to measure GRP78, xBP1s, ATF4, and CHOP composition in cell culture experiments conducted on MDA-MB-468 cell lines.
