# Supplementary material for: Non-invasive electromagnetic fields induce anti-tumor neutrophil reprogramming and enhance checkpoint blockade in breast cancer
Source: Front Immunol. 2026 Oct 16;17:1861048. doi: 10.3389/fimmu.2026.1861048 (PMC13720674; doi:10.3389/fimmu.2026.1861048)
Supplement: Supplementary file 10 [file DataSheet8.pdf]

A)

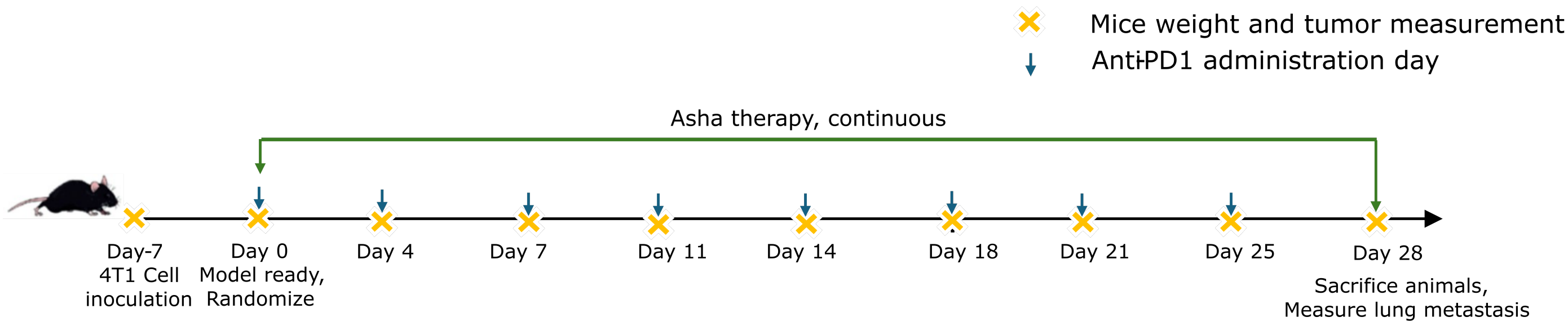

B)

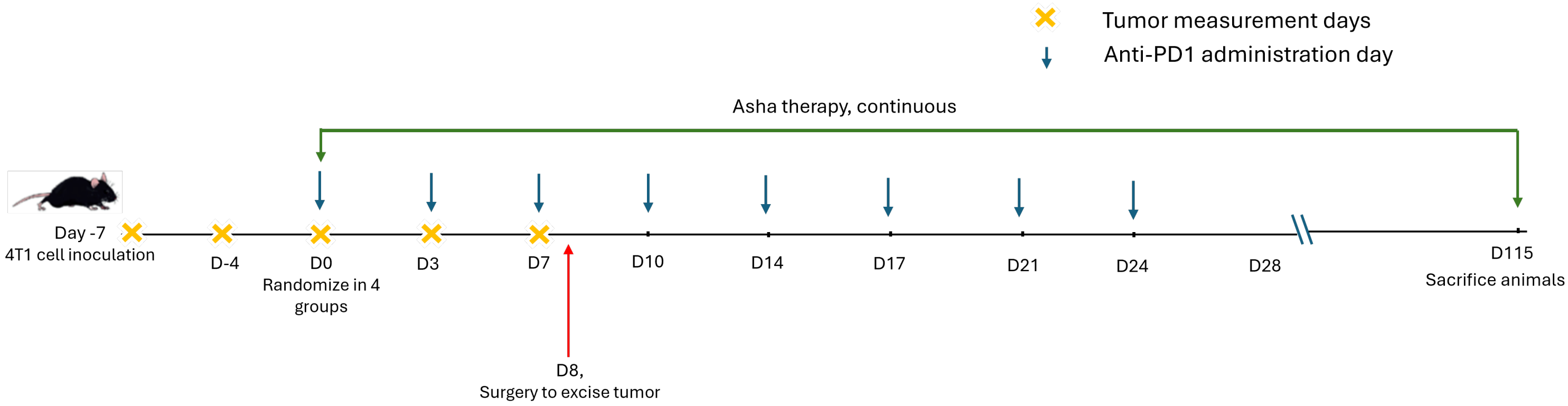

Supplementary Figure S2: A) Schematic representation of the experimental timeline for the orthotopic 4T1 lung metastasis study. B) Schematic representation of the experimental timeline for the orthotopic 4T1 survival study.
