# Supplementary material for: Non-invasive electromagnetic fields induce anti-tumor neutrophil reprogramming and enhance checkpoint blockade in breast cancer
Source: Front Immunol. 2026 Oct 16;17:1861048. doi: 10.3389/fimmu.2026.1861048 (PMC13720674; doi:10.3389/fimmu.2026.1861048)
Supplement: Supplementary file 11 [file DataSheet9.pdf]

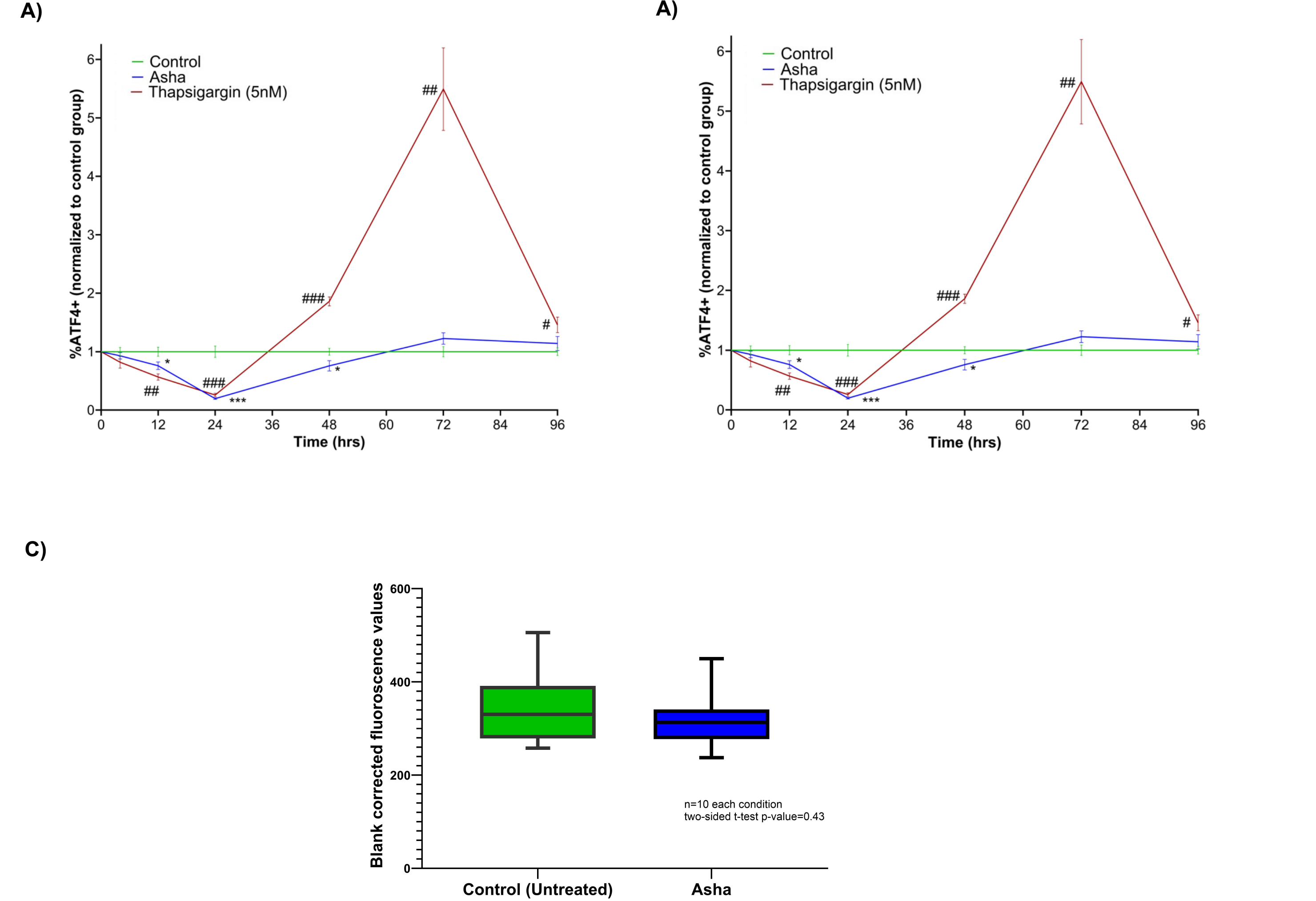

Supplementary Figure S3: A-B) Time-course analysis of ER stress markers in MDA-MB-468 cells exposed to Asha therapy, thapsigargin (5 nM, positive control), or left untreated (control) for up to 96 hours. Levels of CHOP (A) and ATF4 were measured by flow cytometry. Data are presented as mean ± SEM (n=6 per group per time point), normalized to the control group at each time point. Statistical significance was determined using multiple unpaired t-tests comparing each treatment group to the control group at each time point. Significance markers: *, # ($p < 0.05$); **, ## ($p < 0.01$); ***, ### ($p < 0.001$) C) MDA-MB-468 cells exposed to Asha therapy for 96 hours showed no significant reduction in viability compared to untreated controls, as measured by PrestoBlue™ fluorescence assay (p=0.43, two-sided t-test, n=10 per group). Box plots show median, interquartile range, and full range. Values are background-corrected.
